# Use of a Virtual Reality Simulator for Tendon Repair Training: Randomized Controlled Trial

**DOI:** 10.2196/27544

**Published:** 2021-07-12

**Authors:** Tsz-Ngai Mok, Junyuan Chen, Jinghua Pan, Wai-Kit Ming, Qiyu He, Tat-Hang Sin, Jialin Deng, Jieruo Li, Zhengang Zha

**Affiliations:** 1 Institute of Orthopedics Diseases and Center for Joint Surgery and Sports Medicine The First Affiliated Hospital of Jinan University Guangzhou China; 2 Department of General Surgery The First Affiliated Hospital of Jinan University Guangzhou China; 3 Department of Public Health and Preventive Medicine School of Medicine Jinan University Guangzhou China; 4 Pediatric Cardiac Surgery Centre Fuwai Hospital, National Centre for Cardiovascular Diseases, Chinese Academy of Medical Sciences Peking Union Medical College Beijing China

**Keywords:** virtual reality simulators, tendon suture, medical education

## Abstract

**Background:**

Virtual reality (VR) simulators have become widespread tools for training medical students and residents in medical schools. Students using VR simulators are provided with a 3D human model to observe the details by using multiple senses and they can participate in an environment that is similar to reality.

**Objective:**

The aim of this study was to promote a new approach consisting of a shared and independent study platform for medical orthopedic students, to compare traditional tendon repair training with VR simulation of tendon repair, and to evaluate future applications of VR simulation in the academic medical field.

**Methods:**

In this study, 121 participants were randomly allocated to VR or control groups. The participants in the VR group studied the tendon repair technique via the VR simulator, while the control group followed traditional tendon repair teaching methods. The final assessment for the medical students involved performing tendon repair with the “Kessler tendon repair with 2 interrupted tendon repair knots” (KS) method and the “Bunnell tendon repair with figure 8 tendon repair” (BS) method on a synthetic model. The operative performance was evaluated using the global rating scale.

**Results:**

Of the 121 participants, 117 participants finished the assessment and 4 participants were lost to follow-up. The overall performance (a total score of 35) of the VR group using the KS method and the BS method was significantly higher (*P*<.001) than that of the control group. Thus, participants who received VR simulator training had a significantly higher score on the global rating scale than those who received traditional tendon repair training (*P*<.001).

**Conclusions:**

Our study shows that compared with the traditional tendon repair method, the VR simulator for learning tendon suturing resulted in a significant improvement of the medical students in the time in motion, flow of operation, and knowledge of the procedure. Therefore, VR simulator development in the future would most likely be beneficial for medical education and clinical practice.

**Trial Registration:**

Chinese Clinical Trial Registry ChiCTR2100046648; http://www.chictr.org.cn/hvshowproject.aspx?id=90180

## Introduction

The incidence of tendon rupture has been increasing owing to the increasing number of people participating in recreational and competitive sports [1]. Various factors such as the intensity of exercise, overuse, genetic predisposition, and aging can cause tendon rupture [2-4]. Ruptured tendons can have delayed recovery and a high frequency of recurrence [5,6]. Tendon repair is one of the most commonly used techniques in the orthopedics field [7]. Accuracy and proficiency in the suture technique is a fundamental surgical skill that will directly influence the result of the performed operation [8]. Medical students should master both basic and practical suturing concepts to perform an outstanding operation [9]. The standardized goal for flexor tendon repair is to obtain sufficient tensile strength and to facilitate a better recovery [10]. In addition, the repair will allow early mobilization, prevent adhesion formation [11], stimulate tendon healing [12], and improve clinical outcomes. Therefore, tendon repair is an essential skill in the orthopedics field [13,14]. Tendon repair and suture techniques are part of the orthopedic course during clinical training. In traditional training, students only have limited time to practice owing to cost limitations [15,16]. During clinical practice, a real patient is involved at every step in the process. Because of the lack of practical training, medical students need to be guided through a long learning curve to become independent in clinical practice [17].

In recent years, the use of virtual reality (VR) simulators has become widespread in medical school, and VR simulators are promoted for both medical students and resident training [18]. VR simulators for orthopedics provide a holistic learning application, which produces a close-up surgical training experience. Students are provided with a 3D human model to directly observe human details from the point of view of multiple senses, including vision and hearing, and they can participate in an environment relatively close to reality. The use of virtual practical teaching content expands and enriches teaching quality and content [19]. In addition, the use of a VR simulator is a skill that requires time and practice to master. Skills involved in using a simulator include adjusting the master controls, endo wrists, camera navigation, and the requirement for students to picture the VR simulator environment in reality [20]. Recent studies have applied VR simulators in surgical training [21-23], but the aim of this study was to promote a new approach consisting of a shared and independent study platform for medical student education. This study compared traditional tendon repair training with VR simulation of tendon repair and evaluated future applications of VR simulation in the academic medical field.

## Methods

### Study Design

This study is a parallel-design randomized controlled trial comparing VR and control groups. This study was approved by the ethics committee of the First Affiliated Hospital of Jinan University and registered in the Chinese Clinical Trial Registry (Registry: ChiCTR2100046648). Information was collected from all participants after obtaining written informed consent in accordance with the Declaration of Helsinki. All participants were required to complete the final assessment, which was performing tendon repair on synthetic models with 2 different knots, that is, the “Kessler tendon repair with 2 interrupted tendon repair knots” and the “Bunnell tendon repair with figure 8 tendon repair” (KS and BS methods, respectively). The CONSORT checklist was used for this trial.

### Participants

Senior medical students were the eligible participants in this study. They were required to complete the following fundamental courses before entering the randomized control trial: (1) human anatomy, (2) physiology, (3) biochemistry, (4) pathology, (5) pathophysiology, (6) diagnostics, (7) internal medicine, (8) orthopedics, (9) surgical probation, and (10) other professional basic clinical courses. This study excluded any participant who did not meet the above requirements. Written informed consent with a clearly stated study plan was given to all participants. The purpose of this trial was explained to the participants. After informed consent had been signed, we asked the medical students to perform tendon repair on synthetic simulations. A baseline score was given by an orthopedic specialist. Other baseline information, including gender, age, and grade point average, was collected from the medical school database.

### Allocation

**Figure 1:**
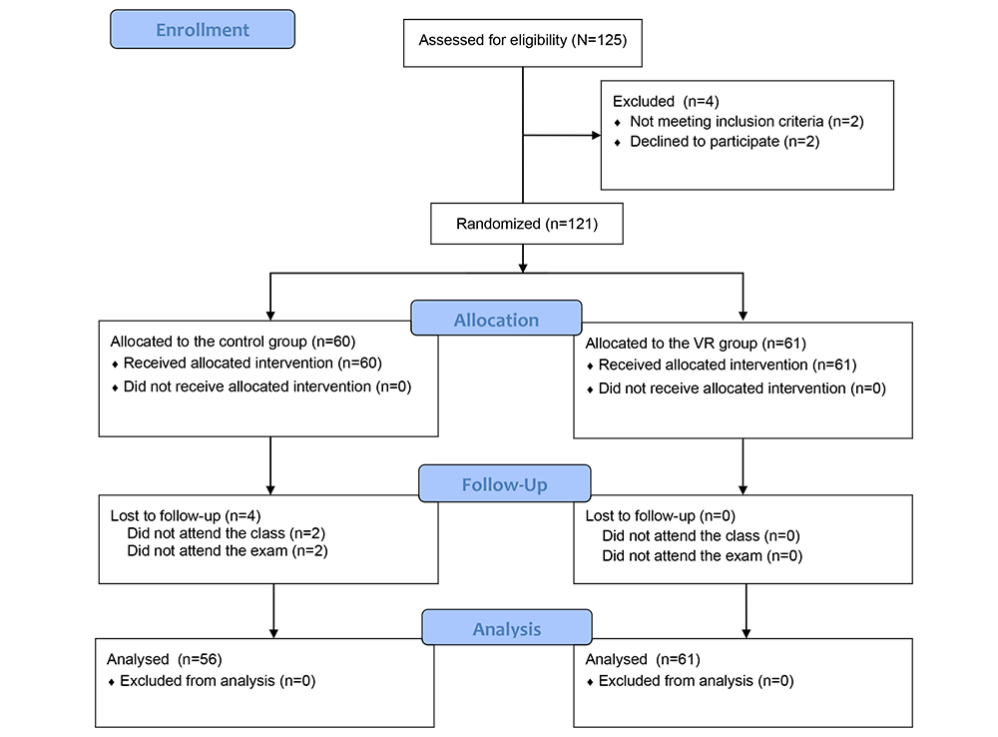
CONSORT 2010 flow diagram showing the study process. VR: virtual reality.

All participants provided written, informed, and oral independently witnessed consent to participate in the research study. A random allocation sequence was generated using a random number table. A sequence was used to allocate the groups of participants to the VR and control groups. For the examination, the students performed tendon repair on a synthetic model. All participants were randomly assigned to one of the two groups. Participants in the VR group (n=61) learned the technique of tendon repair through the VR simulator method, whereas the control group (n=60) used the traditional tendon repair teaching method. The examiners were well-trained surgeons and unaffiliated with the medical school; they evaluated and assigned a score to each final product immediately without knowing the allocation list in a nonbiased manner during preintervention and postintervention assessments. In order to ensure the rigor of the examination, we included a short training session for the examiners. Training helps to clarify the examiner’s role, required behavior, review the marking guidance, marking assignment to standardize the exam, and encourage the consistency of the examiner's marking behavior. At the end of the training, examiners also did a marking exercise to scrutinize examiners' marking behavior. During the examination, medical students were asked not to tell the examiner which group they were assigned to (Figure 1).

### Interventions

The control group participants were required to participate in complete 8 hours of lectures and a 6-hour practical class in medical school for 2 weeks. The participants learned about traumatic orthopedic theory and the fundamentals of tendon repair during the lectures. They practiced tendon repair on synthetic models under the professor’s guidance. In the practice class, students were given a PowerPoint presentation, which provided illustrations, photographs, and step-by-step instructions. They were instructed to review the training material for 1 hour. The VR group participants were required to take the same course as the control group, except for the guided PowerPoint review part. Instead, they practiced with VR simulators (including the VR version and the personal computer [PC] version) for 1 hour in class. The medical students practiced under guidance with detailed instructions. The VR simulator focusses on every participant’s performance while performing tendon repair. The operation in the VR simulator is divided into practice and examination modes. Corresponding notes for each step during the practice mode were provided; however, no notes were provided for the examination mode (Multimedia Appendix 1). The students were required to finish all the required learning in the practice mode before entering the examination mode for assessment. For the VR training section, while half of the students were practicing in the VR simulator, the rest of the students were practicing on the PC version in the training center. These students shifted the training modes after 30 minutes of VR training (see Table 1). All trainings were performed within the classes, and both groups had exactly the same opportunity for practice time.

**Table 1:** Illustration of the trainings undertaken by each group.

Trainings	Virtual reality group	Control group
Lectures (total 8 h)	✓	✓
Practical class (total 6 h)	✓	✓
Guided PowerPoint Review (within the practical class)		✓
Virtual reality + personal computer practice (within the practical class)	✓	
Virtual reality + personal computer assessment (within the practical class)	✓	

### The VR Platform

**Figure 2:**
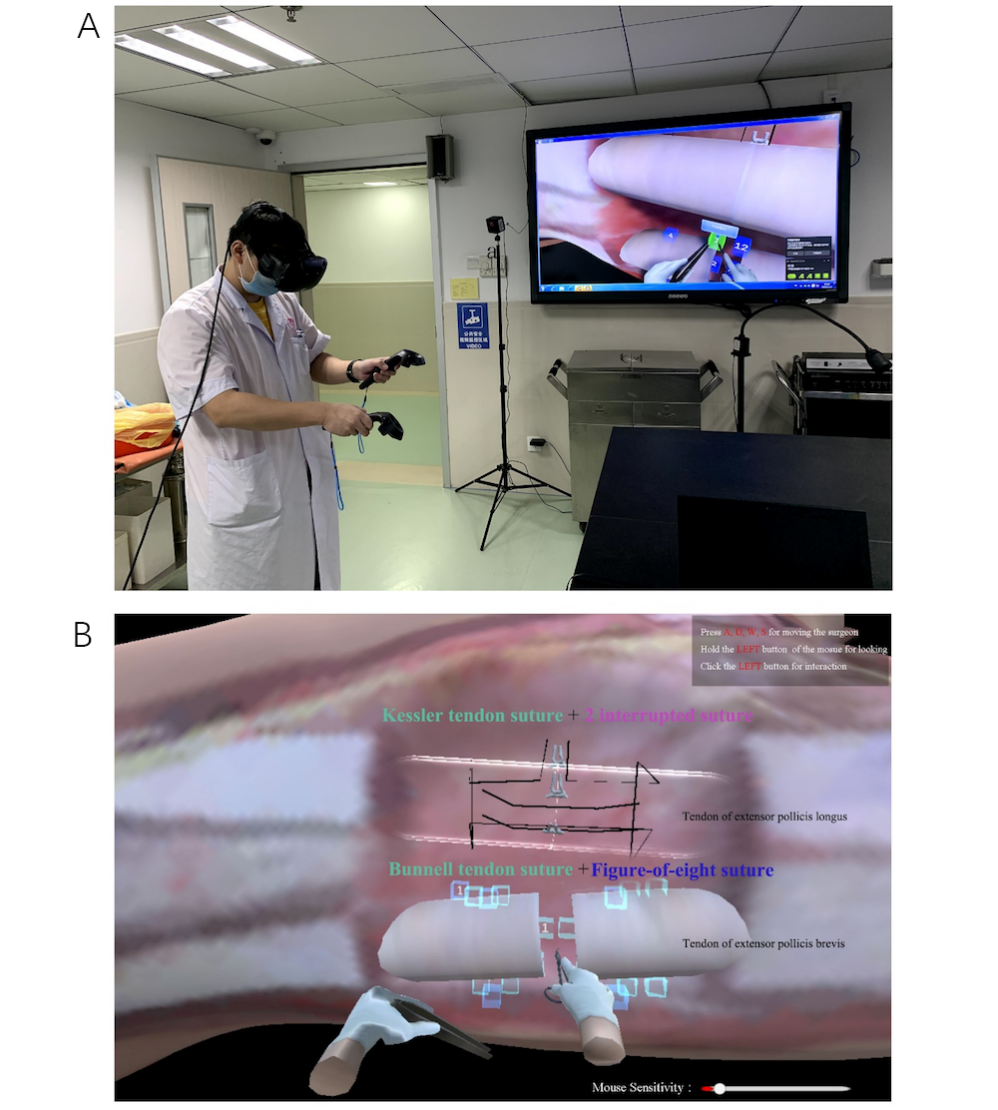
(A) A medical student practicing with a virtual reality simulator; (B) the examination mode for the Kessler and Bunnell tendon suture modes.

The VR simulator used in this study was created by Jinan University and the Department of Orthopedic Surgery and Sports Medicine Center [24]. All VR simulators were classified as HTC Vive VR [25], and the software was SteamVR (JinKe Lu) [26]. The VR simulator method used in this study included an independent study section in which each participant was required to study all the theories on the VR simulator website. This website is open to the public after registration on the website. Two versions are available (ie, PC and VR simulator versions). Students were able to learn the method of tendon repair by controlling the keyboard and mouse in the PC version. Although the PC version is on a computer, a 360° scenario to enhance the study environment is still included. The VR simulator version requires the student to have the HTC Vive VR controller to practice repairing a tendon. Both versions were open to students for practice according to their needs. In addition, the practical study section of tendon repair is performed using the VR simulator with individual steps for each procedure (comprising 7 steps in total). The website provides a section in which students and teachers can communicate and share ideas with each other. This program allows the students to learn in-depth while enhancing their learning through the communication section of the VR simulator. This web-based discussion can overcome the barriers of time and distance. In addition, we measured the effectiveness of this platform, including the PC and VR versions. Because the whole training package was considered, the VR group students were divided into VR and PC groups (Figure 2).

### Performance and Assessment

Both the control and VR groups participated in the research study for 14 days. The results were calculated using the global rating scale. Seven dimensions were incorporated into the tool. The global rating scale shows different aspects of the operative performance. This technology was compared with the traditional teaching method by using the global rating scale for several aspects: (1) repair with respect to tissue, (2) time in motion, (3) instrument handling, (4) tendon repair skill, (5) flow of operation, (6) knowledge of procedure and final suture, and (7) qualitative and objective assessment of all tendon repair performances [27,28]. Each column was scored on a 5-point scale. Explicit descriptors were designed to guide the examiner when evaluating the students’ performance. Each item was scored from 1 (poor performance) to 5 (good performance). Higher scores on the 35-point global rating scale in the final assessment indicated that the participants using the specific method had better performance with respect to the tendon suturing technique [27]. The global rating scale is widely used in the evaluation of surgical behavior, including objective and subjective criteria [29]. It has been used for various types of surgical evaluations such as arthroscopic surgery [30], endoscopic surgery [31], pediatric surgery [32], and orthopedic surgery [33]. Additionally, it has been validated for use with VR systems because it measures nontechnical cognitive skills such as decision making and judgment [34].

### Statistical Analysis

Data were analyzed using the SPSS 23.0 (IBM Corp) software package [35]. The baseline information, including age and grade point average, was analyzed using the independent two-tailed *t* test for parametric data [36]. Differences in the objective and semiobjective measurements between the 2 groups were analyzed using the Mann-Whitney *U* test for nonparametric data [37]. The level of agreement between the semiobjective assessments made by the 2 experts was estimated by the Cohen *k* coefficient. *P* values less than .05 were considered significant [38].

## Results

Between August 1, 2019, and August 12, 2020, 121 potential participants were assessed for study participation in the Medical School of Jinan University. Four participants from the control group dropped out of the program for personal reasons. All participants were required to undergo a final assessment on synthetic models, and the overall score sheet was used to calculate the results. This study analyzed all participants by using the global rating scale described above. The global rating scale baseline is shown for assessing tendon repair differences in the control and VR groups (Table 2). A comparison of the participants in both groups according to age, gender, grade point average, and pretest evaluation revealed no educationally relevant or significant differences. The follow-up ended on September 30, 2020.

Posttraining scores on the global rating scale were used to assess tendon repair by the two groups. Table 3 shows a comparison of the global rating scale scores between the KS and BS methods.

With respect to tissue, no significant difference was found between the VR and control groups using the KS method (*P*=.22) and the BS method (*P*=.21). Participants in the VR group showed higher scores than the control group for time in motion (*P*<.001) for the KS method and the BS method, thereby indicating that the VR group produced better results for time in motion. Regarding instrument handling, no significant difference was found between the VR and control groups for either the KS or the BS method (KS and BS methods *P*=.31 and .16, respectively). With respect to suture skill, the VR group performed better than the control group by using the BS method than the KS method (*P*<.001). In the flow of operation, the VR group performed better than the control group with the KS and BS methods (*P*<.001). With respect to procedure knowledge, the VR group performed better than the control group when performing the KS method (*P*<.001) and the BS method (*P*<.001). The KS method (*P*=.048) and the BS method (*P*<.001) yielded significant results in the final product. Thus, the overall performance of the VR group was significantly better (*P*<.001) than that of the control group with both KS and BS methods (*P*<.001).

**Table 2:** Baseline characteristics.

Characteristics	Control group (n=56)	Virtual reality group (n=61)	*P* value
Age (years), mean (SD)	23.07 (0.97)	22.93 (1.01)	.46
Gender (male), n (%)	24 (43)	31 (51)	.39
Grade point average, mean (SD)	3.02 (0.54)	3.19 (0.49)	.66
Kessler tendon repair with 2 interrupted tendon repair knots, median (IQR)	8.00 (7-9)	8.00 (7-9)	.13
Bunnell tendon repair with figure 8 tendon repair, median (IQR)	8.00 (7-9)	8.00 (7-9)	.25

**Table 3:** Posttraining scores on the global rating scale to assess tendon repair.^a^

Repair method, aspects considered, global rating scale score (1: poor, 5: good)							Control group (n=56)		Virtual reality group (n=61)		*P* value
**Kessler tendon repair with 2 interrupted tendon repair knots**											
	**With respect to tissue, n (%)**										.22
				1		0 (0)		8 (13)			
				2		3 (5)		41 (67)			
				3		52 (93)		10 (16)			
				4		1 (2)		2 (3)			
				5		0 (0)		0 (0)			
	**Time in motion, n (%)**										*<.001*
				1		2 (4)		0 (0)			
				2		14 (25)		0 (0)			
				3		23 (41)		20 (33)			
				4		17 (30)		40 (66)			
				5		0 (0)		1 (2)			
	**Instrument handling, n (%)**										.31
				1		0 (0)		0 (0)			
				2		8 (14)		13 (21)			
				3		42 (75)		43 (71)			
				4		6 (11)		5 (8)			
				5		0 (0)		0 (0)			
	**Suture skill, n (%)**										.05
				1		0 (0)		0 (0)			
				2		11 (20)		6 (10)			
				3		36 (64)		38 (62)			
				4		9 (16)		14 (23)			
				5		0 (0)		3 (5)			
	**Flow of operation, n (%)**										*<.001*
				1		0 (0)		0 (0)			
				2		19 (34)		0 (0)			
				3		33 (59)		11 (18)			
				4		4 (7)		49 (80)			
				5		0 (0)		1 (2)			
	**Knowledge of procedure, n (%)**										*<.001*
				1		0 (0)		0 (0)			
				2		15 (27)		1 (2)			
				3		41 (73)		1 (2)			
				4		0 (0)		42 (69)			
				5		0 (0)		17 (28)			
	**Final product, n (%)**										.048
				1		0 (0)		0 (0)			
				2		3 (5)		12 (20)			
				3		49 (88)		24 (39)			
				4		3 (5)		22 (36)			
				5		1 (2)		3 (5)			
	Overall performance, median (range)						20 (18-22)		24 (21-29)		*<.001*
**Bunnell tendon repair with figure 8 tendon repair**											
		**With respect to** **tissue, n (%)**									.21
			1		0 (0)			0 (0)			
			2		15 (27)			23 (38)			
			3		41 (73)			38 (62)			
			4		0 (0)			0 (0)			
			5		0 (0)			0 (0)			
		**Time in motion, n (%)**									*<.001*
			1		0 (0)			0 (0)			
			2		15 (27)			5 (8)			
			3		41 (73)			32 (53)			
			4		0 (0)			23 (74)			
			5		0 (0)			1 (2)			
		**Instrument handling, n (%)**									.16
			1		0 (0)			0 (0)			
			2		8 (14)			15 (25)			
			3		32 (57)			33 (54)			
			4		16 (29)			13(21)			
			5		0 (0)			0 (0)			
		**Suture skill, n (%)**									*<.001*
			1		0 (0)			0 (0)			
			2		17 (30)			10 (16)			
			3		39 (70)			30 (49)			
			4		0 (0)			21 (34)			
			5		0 (0)			0 (0)			
		**Flow of operation, n (%)**									*<.001*
			1		0 (0)			0 (0)			
			2		27 (48)			1 (2)			
			3		25 (45)			26 (43)			
			4		0 (0)			28 (46)			
			5		4 (7)			6 (10)			
		**Knowledge of procedure, n (%)**									*<.001*
			1		0 (0)			0 (0)			
			2		21 (38)			2 (3)			
			3		21 (38)			11 (18)			
			4		14 (25)			47 (77)			
			5		0 (0)			1 (2)			
		**Final product, n (%)**									*<.001*
			1		0 (0)			0 (0)			
			2		8 (14)			11 (18)			
			3		41 (73)			31 (51)			
			4		1 (2)			9 (15)			
			5		6 (11)			10 (16)			
		Overall performance, median (range)			20.00 (16-23)			23.00 (20-26)		*<.001*	

^a^Data in italics indicate significant differences. Both the methods were assessed using the 35-point global rating scale.

## Discussion

### Principal Results

Several surgical trainings involve composite models, VR simulation, cognitive task analysis, and cadaver specimens. VR-simulated studies for surgical practice, such as laparoscopic, cardiovascular, and arthroscopic surgeries, can be found in the literature [39-41]. In the field of orthopedics, researchers mainly focus on sophisticated surgical procedures, for example, (thoracic) pedicle screw placement and insertion (lateral mass screw placement) [42-44], (percutaneous) vertebroplasty [45], knee arthroscopy [38], and hip arthroplasty [46]. These procedures are performed by surgeons who are specialized in orthopedics. In medical schools, for every general practice physician, standard tendon repair is of prime importance in fundamental surgical skills. Thus, it is necessary to develop tendon repair training [47]. To our knowledge, this is the first study to adopt VR simulation for tendon repair training. Adopting VR simulation in regular curricula is challenging owing to the limited efficacy of VR as a learning tool [48]. To clarify the effectiveness, we demonstrated that the VR simulator was an effective tool in the acquisition of tendon repair in our blinded randomized trial. Modern VR simulations have a common disadvantage, that is, high cost. Clarke [48] reported that individual simulators cost up to 6-figure sums. Our platform removed the cost for the students and was open to the public to maximize cost-effectiveness. To consider whether the VR simulator is an educational tool, the results have to be statistically significant with positive feedback. If the surgical performance of the VR group participants did not improve, the VR simulator was not considered as part of the regular training.

A VR simulator plays a major role in the medical field. The lack of medical practice and uneven distribution of medical resources in various regions has resulted in a decrease in clinical practice opportunities for medical students. In addition to tendon repair studies, the VR simulator can perform simulated surgery. Future surgeons can practice with a VR simulator until they are comfortable performing the operation. In addition, experienced surgeons can also study the simulation aspect of the VR simulator to learn and explore new surgical techniques or to discover other surgical options. The application of virtual technology in medicine, medical education, and clinical treatment will have a major impact on the medical system.

During the COVID-19 pandemic, the use of technology has become a popular topic in the medical education field. Tendon repair is a procedure that requires senior professional surgeons; therefore, medical students and junior doctors may not have sufficient practice to be able to perform suturing independently. A possible solution to this problem is that junior doctors practice using the VR simulator, thereby becoming more familiar with the procedure and more confident when performing it. The VR simulator can maximize a medical student’s efficiency with respect to mastering this technique. It has been proven that the VR simulator in a simulation laboratory rather than in an operating room is a better practice method than the traditional classroom study in terms of the flexibility of location [20]. Medical students or residents can perform tendon repair via the VR simulator before performing a formal operation. Using the VR simulator serves the purpose of shortening the operating time, reducing operation errors, and alleviating patients’ postprocedural pain [49]. However, the expense of textbooks or teaching assistance when using the traditional method has no significant comparison with the investment in equipment for VR simulators.

The VR simulator can provide a realistic surgical scenario, thus allowing students to train for a particular skill continuously or to master any unfamiliar procedure. The findings of our study show that students learning via the VR simulator had significantly better scores than those learning via the traditional method with respect to the tendon repair technique (*P*<.001). This finding may indicate that students using a VR simulator will be able to follow the whole operation more carefully and master the knowledge of the procedure in the future. While we were developing the VR simulator, we tried to mimic reality based on mathematical models by simulating a surgical setting, instruments, training objects, and interactions. We aimed to create an ideal simulator that is realistic in multiple dimensions such as simulation physics, optical properties, and haptic feedback [50]. The system allows students to apply their knowledge and practical skills in a realistic and tactile environment. Another advantage of the VR simulator method is that students can independently perform the surgery, hence increasing the student’s ability to master the technique fully. A VR simulator allows a student to practice at his/her convenience with multiple repetitions, regardless of the availability of cadavers or human trials [51]. Using the VR simulator, the student can maximize his/her surgical efficiency at his/her own learning time even after the scheduled class, which will serve to increase the study’s performance and study result.

A VR simulator can reduce the high costs of conducting animal or human trials [52]. More realistic training usually involves training on animals, but this method is expensive and not available in many medical schools. A VR simulator would provide many benefits in this situation. VR simulators allow students to repeat the exercise several times—an action that can ensure that students are totally familiar with all important concepts before surgery. In addition, students are able to familiarize themselves with the technique before surgery, which also guarantees safety for the patient. Studies show that surgeons using VR and other simulation methods for practicing surgical techniques can reduce operative time or possible errors, thereby increasing their confidence and decreasing uncertainty in the procedure outcomes [53]. In addition, most surveyed residents and directors believe that a surgical simulation is a useful tool for complementing traditional forms of training on animals, cadavers, or synthetic models [51]. Although the texture of operation cannot be simulated exactly on a VR simulator, using animals, cadavers, or synthetic models with a VR simulator could also allow students to practice their techniques with more understanding, hence reducing the cost and availability issue of animals, cadavers, and synthetic models.

Surgical training is different from other courses in medical school because students need to be exposed to nonstandard or beyond standard levels of teaching to help them understand how much they need to learn or improve. Studying with different methods can help students have a clear understanding of this curriculum. Surgical education departments have already purchased some effective training equipment that can help medical students achieve consistent and measurably high level of performance. Therefore, combining traditional and alternative teaching methods such as composite models and cognitive task analysis is necessary for the future [54]. Al-Nammari et al [55] found that the average duration of orthopedic education in medical schools in the United Kingdom was approximately 2.65 weeks; however, medical schools may not provide their students with enough scheduled course time to master musculoskeletal medicine. One of the solutions to this problem is that students combine VR simulation with traditional methods, which will save time and maximize students’ efforts in musculoskeletal medicine if the student is interested in this field. This study also shows that students with interest in orthopedics had a statistically better understanding of musculoskeletal medicine than their counterparts. In a broader view, more elite students entering the field of musculoskeletal medicine will increase the possibility of new discoveries in musculoskeletal medicine.

VR simulators have good reusability. Once a VR simulator is established, the study will not be limited to that particular location or the number of students. The VR software can be freely downloaded by students from the internet. Therefore, medical students can study in any location and are not limited to the classroom or training room. Rather than taking turns practicing on subjects, students are able to practice together using the VR simulator. The 3 important advantages of students using VR simulators concurrently are as follows: (1) VR simulators allow students to perform the experiment together, (2) VR simulators allow the exchange of ideas or knowledge of a topic at the same level, and (3) VR simulators ensure that everyone is on schedule for the course.

VR simulators will be part of the future technology in the medical field, and medical students should be exposed to this technology as soon as possible to familiarize themselves with this method. In addition, feedback from students using a VR simulator can help improve the simulator, and further developments may be performed. The application of virtual technology in the field of medicine, medical education, and clinical treatment will have a major impact on health care and the development of the medical field and on the training of young doctors. Both software and hardware in the VR simulator can be flexibly configured according to the needs of teaching. In addition to tendon repair, VR simulators provide a set of virtual instruments that can be developed into different functions. The scope of virtual orthopedics is very wide and includes the fields of endoscopic protection, radiosurgery, microsurgery, and remote surgery. Moreover, a VR simulator is currently used in neurosurgery, eye, heart, plastic, and abdominal surgeries, and many other areas. Therefore, the widespread use of a VR simulator indicates that studying or performing surgery via a VR simulator is an effective method for completing this training task.

This study is the first to show that a VR simulator can also be applicable in medical schools and that it is a very effective method for medical students to enhance their learning through multiple repetitions by participating in a 360° VR environment. In addition, a VR simulator should be used in sections not only for complex surgeries but also in important presurgical sections, for example, tendon suturing. Studies have shown that medical students enjoy early exposure to clinical practice and perceive that it is valuable for future studies [56,57]. As mentioned earlier, multiple medical professions have attempted to utilize VR simulators for their medical training. However, most professions cannot overcome a critical issue—the high cost. This high cost also results in only using the compatible device of the VR simulator to minimize costs. The VR simulator program was uploaded on the internet, and all medical students could choose the versions according to their own needs and the available equipment. The PC version can be downloaded on the internet at no cost. This inexpensive, remote, detailed, sustainable, and effective platform should be used in medical schools for all medical students. A successful education system should be accessible to all students [58]. This VR simulator with high accessibility allows different students to have multiple practice opportunities and to participate in in-depth discussions in the specific communication section. Designing this program was costly; however, with more students using this program, the cost-effectiveness of this system can maximized.

Despite the benefits of using VR simulators to train medical students, there are some challenges for medical schools to adopt this new approach in the future. For designing the program, the development corporation or the program writers who are not medical professionals have limited concepts or knowledge of the orthopedic procedure. This may lead to simulation results of nondetailed procedures, and medical students may miss the surgical details when practicing surgery in VR simulators. To overcome this obstacle, we had extensive discussions and we shared opinions back and forth between the medical school and the development team during the early period of establishing the VR simulator. It took at least a year to achieve an acceptable VR simulator for medical students. The other problem of VR simulators is the lack of validated score measures. Therefore, the final assessment was evaluated on synthetic models by using the global rating scale. Surgical training is a comprehensive process; therefore, medical students and surgeons may take years to master a surgical technique. The VR simulator is part of the comprehensive training but not the only training technique. Our findings show that the VR simulator is an effective method among the traditional teaching methods. We hope that future studies can focus on an effective VR simulator measurement scale or comparative evaluations between simulators.

### Limitations

The follow-up period in this study can only reflect the short-term effect of the VR simulator, which was a limitation of this study. The long-term effects on orthopedic specialists who practice on VR simulators could take years to evaluate.

### Conclusion

Compared to the traditional tendon repair method, the VR simulator for learning tendon repair significantly improves medical students’ time in motion, suture skill, the flow of operation, and knowledge of the surgical procedure. Our paper sets an example for future VR simulator development for medical curricula.

## Appendix

Multimedia Appendix 1 Virtual reality simulator showing every step of the suturing process.

Multimedia Appendix 2 CONSORT-eHEALTH checklist (V 1.6.1).

## Abbreviations

BS: Bunnell tendon repair with figure 8 tendon repair
KS: Kessler tendon repair with 2 interrupted tendon repair knots
PC: personal computer
VR: virtual reality
