# Supplementary material for: Use of a Virtual Reality Simulator for Tendon Repair Training: Randomized Controlled Trial
Source: JMIR Serious Games. 2021 Jul 12;9(3):e27544. doi: 10.2196/27544 (PMC8314161; doi:10.2196/27544)
Supplement: Multimedia Appendix 1 [file games_v9i3e27544_app1.docx]

Appendix 1: In this VR simulator, participant will learn each step in suturing process:

| Suturing Step (#) in VR simulator | # of stitches | Additional requirement | Detail Procedure |
| --- | --- | --- | --- |
| 1 | 1 | ---- | Expose the two stumps of the extensor pollicis longus tendon, and pass the needle parallel to the tendon from the stump side of the tendon (8-10mm from the stump). Next step is to go diagonally out on the same side (30cm long 3"0" tendon suture). |
| 2 | 2-3 | ---- | Position the needle horizontally through the tendon at 2-3mm where the needle exits the tendon. Then place the needle 2-3mm below the place where the tendon exits the needle. Advance the obliquely needle slightly, and then pass the parallel tendon through the section of the tendon; |
| 3 | 4-5 | ---- | Insert the needle into the other trimmed tendon stump parallel to the tendon, obliquely pass it out on the same side (8-10mm from the stump), and then tighten the suture to break the tendon. (as shown in Fig1.) |
| 4 | 6 | ---- | pass the needle horizontally through the tendon at 2-3mm above the needle exit point, and then place the needle 2-3mm below the tendon exit point. Insert the obliquely needle slightly, and then parallel the tendon through the cross section of the tendon; |
| 5 | -- | Knotting and trimming | Draw the suture again, make the two broken ends close and tie the knot, trim the thread; |
| 6 | -- | Strengthen the suture | Suture one stitch on both sides of the sutured end to reinforce and tie the knot; |
| 7 | -- | Suture the extensor pollicis brevis tendon | Repeat steps 1-6 to suture the extensor pollicis brevis tendon. |
