# Supplementary material for: Use of a Virtual Reality Simulator for Tendon Repair Training: Randomized Controlled Trial
Source: JMIR Serious Games. 2021 Jul 12;9(3):e27544. doi: 10.2196/27544 (PMC8314161; doi:10.2196/27544)
Supplement: Multimedia Appendix 2 [file games_v9i3e27544_app2.pdf]

CONSORT-EHEALTH (V 1.6.1) - Submission/Publication Form

The CONSORT-EHEALTH checklist is intended for authors of randomized trials evaluating web-based and Internet-based applications/interventions, including mobile interventions, electronic games (incl multiplayer games), social media, certain telehealth applications, and other interactive and/or networked electronic applications. Some of the items (e.g. all subitems under item 5 - description of the intervention) may also be applicable for other study designs.

The goal of the CONSORT EHEALTH checklist and guideline is to be
a) a guide for reporting for authors of RCTs,
b) to form a basis for appraisal of an ehealth trial (in terms of validity)

CONSORT-EHEALTH items/subitems are MANDATORY reporting items for studies published in the Journal of Medical Internet Research and other journals / scientific societies endorsing the checklist.

Items numbered 1., 2., 3., 4a., 4b etc are original CONSORT or CONSORT-NPT (non-pharmacologic treatment) items.
Items with Roman numerals (i., ii, iii, iv etc.) are CONSORT-EHEALTH extensions/clarifications.

As the CONSORT-EHEALTH checklist is still considered in a formative stage, we would ask that you also RATE ON A SCALE OF 1-5 how important/useful you feel each item is FOR THE PURPOSE OF THE CHECKLIST and reporting guideline (optional).

Mandatory reporting items are marked with a red *.
In the textboxes, either copy & paste the relevant sections from your manuscript into this form - please include any quotes from your manuscript in QUOTATION MARKS,
or answer directly by providing additional information not in the manuscript, or elaborating on why the item was not relevant for this study.

YOUR ANSWERS WILL BE PUBLISHED AS A SUPPLEMENTARY FILE TO YOUR PUBLICATION IN JMIR AND ARE CONSIDERED PART OF YOUR PUBLICATION (IF ACCEPTED).
Please fill in these questions diligently. Information will not be copyedited, so please use proper spelling and grammar, use correct capitalization, and avoid abbreviations.

DO NOT FORGET TO SAVE AS PDF _AND_ CLICK THE SUBMIT BUTTON SO YOUR ANSWERS ARE IN OUR DATABASE !!!

Citation Suggestion (if you append the pdf as Appendix we suggest to cite this paper in the caption):
Eysenbach G, CONSORT-EHEALTH Group
CONSORT-EHEALTH: Improving and Standardizing Evaluation Reports of Web-based and Mobile Health Interventions
J Med Internet Res 2011;13(4):e126
URL: http://www.jmir.org/2011/4/e126/

doi: 10.2196/jmir.1923
PMID: 22209829

*必填

Your name *

First Last

Jieruo Li

Primary Affiliation (short), City, Country *

University of Toronto, Toronto, Canada

Jinan University, Guangzhou, China

Your e-mail address *

abc@gmail.com

ilorugaie@163.com

Title of your manuscript *

Provide the (draft) title of your manuscript.

A New Approach to Tendon Repair Training: Virtual Reality Simulator

Name of your App/Software/Intervention *

If there is a short and a long/alternate name, write the short name first and add the long name in brackets.

VR platform

Evaluated Version (if any)

e.g. "V1", "Release 2017-03-01", "Version 2.0.27913"

您的答案

Language(s) *

What language is the intervention/app in? If multiple languages are available, separate by comma (e.g. "English, French")

Chinese

URL of your Intervention Website or App

e.g. a direct link to the mobile app on app in appstore (itunes, Google Play), or URL of the website. If the intervention is a DVD or hardware, you can also link to an Amazon page.

http://www.ilab-x.com/details?id=2934&isView=true

URL of an image/screenshot (optional)

http://www.ilab-x.com/details?id=2934&isView=true

Accessibility *

Can an enduser access the intervention presently?

- (x) access is free and open
- ( ) access only for special usergroups, not open
- ( ) access is open to everyone, but requires payment/subscription/in-app purchases
- ( ) app/intervention no longer accessible
- ( ) 其他：

Primary Medical Indication/Disease/Condition *

e.g. "Stress", "Diabetes", or define the target group in brackets after the condition, e.g. "Autism (Parents of children with)", "Alzheimers (Informal Caregivers of)"

Tendon Rupture

Primary Outcomes measured in trial *

comma-separated list of primary outcomes reported in the trial

Global rating scale (GRS) on “Kessler tendon r

Secondary/other outcomes

Are there any other outcomes the intervention is expected to affect?

Global rating scale (GRS) on “Bunnell tendon repair with figure 8 tendon repair”

Recommended "Dose" *

What do the instructions for users say on how often the app should be used?

- (●) Approximately Daily
- ( ) Approximately Weekly
- ( ) Approximately Monthly
- ( ) Approximately Yearly
- ( ) "as needed"
- ( ) 其他：

Approx. Percentage of Users (starters) still using the app as recommended after 3 months *

- ○ unknown / not evaluated
- ○ 0-10%
- ○ 11-20%
- ○ 21-30%
- ○ 31-40%
- ◉ 41-50%
- ○ 51-60%
- ○ 61-70%
- ○ 71%-80%
- ○ 81-90%
- ○ 91-100%
- ○ 其他：

Overall, was the app/intervention effective? *

- ◉ yes: all primary outcomes were significantly better in intervention group vs control
- ○ partly: SOME primary outcomes were significantly better in intervention group vs control
- ○ no statistically significant difference between control and intervention
- ○ potentially harmful: control was significantly better than intervention in one or more outcomes
- ○ inconclusive: more research is needed
- ○ 其他：

Article Preparation Status/Stage *

At which stage in your article preparation are you currently (at the time you fill in this form)

- ○ not submitted yet - in early draft status
- ○ not submitted yet - in late draft status, just before submission
- ○ submitted to a journal but not reviewed yet
- ○ submitted to a journal and after receiving initial reviewer comments
- ◉ submitted to a journal and accepted, but not published yet
- ○ published
- ○ 其他:

Journal *

If you already know where you will submit this paper (or if it is already submitted), please provide the journal name (if it is not JMIR, provide the journal name under "other")

- ○ not submitted yet / unclear where I will submit this
- ○ Journal of Medical Internet Research (JMIR)
- ○ JMIR mHealth and UHealth
- ◉ JMIR Serious Games
- ○ JMIR Mental Health
- ○ JMIR Public Health
- ○ JMIR Formative Research
- ○ Other JMIR sister journal
- ○ 其他:

Is this a full powered effectiveness trial or a pilot/feasibility trial? *

- ◯ Pilot/feasibility
- ◉ Fully powered

Manuscript tracking number *

If this is a JMIR submission, please provide the manuscript tracking number under "other" (The ms tracking number can be found in the submission acknowledgement email, or when you login as author in JMIR. If the paper is already published in JMIR, then the ms tracking number is the four-digit number at the end of the DOI, to be found at the bottom of each published article in JMIR)

- ◯ no ms number (yet) / not (yet) submitted to / published in JMIR
- ◉ 其他: #27544

TITLE AND ABSTRACT

1a) TITLE: Identification as a randomized trial in the title

1a) Does your paper address CONSORT item 1a? *

I.e does the title contain the phrase "Randomized Controlled Trial"? (if not, explain the reason under "other")

- ◯ yes
- ◉ 其他: We wonder if it would be better to change the title as 'A New Virtual R

1a-i) Identify the mode of delivery in the title

Identify the mode of delivery. Preferably use “web-based” and/or “mobile” and/or “electronic game” in the title. Avoid ambiguous terms like “online”, “virtual”, “interactive”. Use “Internet-based” only if Intervention includes non-web-based Internet components (e.g. email), use “computer-based” or “electronic” only if offline products are used. Use “virtual” only in the context of “virtual reality” (3-D worlds). Use “online” only in the context of “online support groups”. Complement or substitute product names with broader terms for the class of products (such as “mobile” or “smart phone” instead of “iphone”), especially if the application runs on different platforms.

	1	2	3	4	5	
subitem not at all important	○	○	○	○	◉	essential

清除選取

Does your paper address subitem 1a-i? *

Copy and paste relevant sections from manuscript title (include quotes in quotation marks "like this" to indicate direct quotes from your manuscript), or elaborate on this item by providing additional information not in the ms, or briefly explain why the item is not applicable/relevant for your study

The title was used the “virtual” only in the context of “virtual reality” (3-D worlds).

1a-ii) Non-web-based components or important co-interventions in title

Mention non-web-based components or important co-interventions in title, if any (e.g., “with telephone support”).

	1	2	3	4	5	
subitem not at all important	◉	○	○	○	○	essential

清除選取

Does your paper address subitem 1a-ii?

Copy and paste relevant sections from manuscript title (include quotes in quotation marks "like this" to indicate direct quotes from your manuscript), or elaborate on this item by providing additional information not in the ms, or briefly explain why the item is not applicable/relevant for your study

No non-web-based components or important co-interventions was applied for the app.

1a-iii) Primary condition or target group in the title

Mention primary condition or target group in the title, if any (e.g., “for children with Type I Diabetes”)
Example: A Web-based and Mobile Intervention with Telephone Support for Children with Type I Diabetes: Randomized Controlled Trial

	1	2	3	4	5	
subitem not at all important	◉	○	○	○	○	essential

清除選取

Does your paper address subitem 1a-iii? *

Copy and paste relevant sections from manuscript title (include quotes in quotation marks "like this" to indicate direct quotes from your manuscript), or elaborate on this item by providing additional information not in the ms, or briefly explain why the item is not applicable/relevant for your study

We have mentioned the title as a surgical training.

1b) ABSTRACT: Structured summary of trial design, methods, results, and conclusions

NPT extension: Description of experimental treatment, comparator, care providers, centers, and blinding status.

1b-i) Key features/functionalities/components of the intervention and comparator in the METHODS section of the ABSTRACT

Mention key features/functionalities/components of the intervention and comparator in the abstract. If possible, also mention theories and principles used for designing the site. Keep in mind the needs of systematic reviewers and indexers by including important synonyms. (Note: Only report in the abstract what the main paper is reporting. If this information is missing from the main body of text, consider adding it)

	1	2	3	4	5	
subitem not at all important	○	○	○	○	◉	essential

清除選取

Does your paper address subitem 1b-i? *

Copy and paste relevant sections from the manuscript abstract (include quotes in quotation marks "like this" to indicate direct quotes from your manuscript), or elaborate on this item by providing additional information not in the ms, or briefly explain why the item is not applicable/relevant for your study

The participants in the VR group studied the tendon repair technique via the VR simulator, while the control group followed traditional tendon suture teaching methods. The final assessment was to perform tendon repair with the “Kessler tendon repair with 2 interrupted tendon repair knots” (KS) method and the “Bunnell tendon repair with figure 8 tendon repair” (BS) method on a synthetic model.

1b-ii) Level of human involvement in the METHODS section of the ABSTRACT

Clarify the level of human involvement in the abstract, e.g., use phrases like “fully automated” vs. “therapist/nurse/care provider/physician-assisted” (mention number and expertise of providers involved, if any). (Note: Only report in the abstract what the main paper is reporting. If this information is missing from the main body of text, consider adding it)

	1	2	3	4	5	
subitem not at all important	◉	○	○	○	○	essential

清除選取

Does your paper address subitem 1b-ii?

Copy and paste relevant sections from the manuscript abstract (include quotes in quotation marks "like this" to indicate direct quotes from your manuscript), or elaborate on this item by providing additional information not in the ms, or briefly explain why the item is not applicable/relevant for your study

the VR stimulator did not involve human involvement

1b-iii) Open vs. closed, web-based (self-assessment) vs. face-to-face assessments in the METHODS section of the ABSTRACT

Mention how participants were recruited (online vs. offline), e.g., from an open access website or from a clinic or a closed online user group (closed usergroup trial), and clarify if this was a purely web-based trial, or there were face-to-face components (as part of the intervention or for assessment). Clearly say if outcomes were self-assessed through questionnaires (as common in web-based trials). Note: In traditional offline trials, an open trial (open-label trial) is a type of clinical trial in which both the researchers and participants know which treatment is being administered. To avoid confusion, use “blinded” or “unblinded” to indicated the level of blinding instead of “open”, as “open” in web-based trials usually refers to “open access” (i.e. participants can self-enrol). (Note: Only report in the abstract what the main paper is reporting. If this information is missing from the main body of text, consider adding it)

	1	2	3	4	5	
subitem not at all important	○	○	○	○	◉	essential

清除選取

Does your paper address subitem 1b-iii?

Copy and paste relevant sections from the manuscript abstract (include quotes in quotation marks "like this" to indicate direct quotes from your manuscript), or elaborate on this item by providing additional information not in the ms, or briefly explain why the item is not applicable/relevant for your study

The final assessment was to perform tendon repair with the “Kessler tendon repair with 2 interrupted tendon repair knots” (KS) method and the “Bunnell tendon repair with figure 8 tendon repair” (BS) method on a synthetic model.

1b-iv) RESULTS section in abstract must contain use data

Report number of participants enrolled/assessed in each group, the use/uptake of the intervention (e.g., attrition/adherence metrics, use over time, number of logins etc.), in addition to primary/secondary outcomes. (Note: Only report in the abstract what the main paper is reporting. If this information is missing from the main body of text, consider adding it)

	1	2	3	4	5	
subitem not at all important	○	○	○	○	◉	essential

清除選取

Does your paper address subitem 1b-iv?

Copy and paste relevant sections from the manuscript abstract (include quotes in quotation marks "like this" to indicate direct quotes from your manuscript), or elaborate on this item by providing additional information not in the ms, or briefly explain why the item is not applicable/relevant for your study

The overall performance (a total score of 35) for the VR group using the “KS method” was significantly higher ($p < 0.001$) than that of the control group. Moreover, for the BS method, the VR group also had a significantly better result ($p < 0.001$) than the control group.

1b-v) CONCLUSIONS/DISCUSSION in abstract for negative trials

Conclusions/Discussions in abstract for negative trials: Discuss the primary outcome - if the trial is negative (primary outcome not changed), and the intervention was not used, discuss whether negative results are attributable to lack of uptake and discuss reasons. (Note: Only report in the abstract what the main paper is reporting. If this information is missing from the main body of text, consider adding it)

	1	2	3	4	5	
subitem not at all important	○	○	○	○	◉	essential

清除選取

Does your paper address subitem 1b-v?

Copy and paste relevant sections from the manuscript abstract (include quotes in quotation marks "like this" to indicate direct quotes from your manuscript), or elaborate on this item by providing additional information not in the ms, or briefly explain why the item is not applicable/relevant for your study

The use of the VR simulator to learn tendon suturing resulted in a significant improvement in the time in motion, flow of operation, and knowledge of procedure by medical students compared with the traditional tendon suture method.

INTRODUCTION

2a) In INTRODUCTION: Scientific background and explanation of rationale

2a-i) Problem and the type of system/solution

Describe the problem and the type of system/solution that is object of the study: intended as stand-alone intervention vs. incorporated in broader health care program? Intended for a particular patient population? Goals of the intervention, e.g., being more cost-effective to other interventions, replace or complement other solutions? (Note: Details about the intervention are provided in “Methods” under 5)

	1	2	3	4	5	
subitem not at all important	○	○	○	○	◉	essential

清除選取

Does your paper address subitem 2a-i? *

Copy and paste relevant sections from the manuscript (include quotes in quotation marks "like this" to indicate direct quotes from your manuscript), or elaborate on this item by providing additional information not in the ms, or briefly explain why the item is not applicable/relevant for your study

The incidence of tendon rupture has been growing due to the increasing number of people participating in recreational and competitive sports [1]. Various factors, such as the intensity of exercise, overuse, genetic predisposition, and ageing, can cause tendon rupture [2-4]. Ruptured tendons can have delayed recovery and a high frequency of recurrence [5, 6].

2a-ii) Scientific background, rationale: What is known about the (type of) system

Scientific background, rationale: What is known about the (type of) system that is the object of the study (be sure to discuss the use of similar systems for other conditions/diagnoses, if appropiate), motivation for the study, i.e. what are the reasons for and what is the context for this specific study, from which stakeholder viewpoint is the study performed, potential impact of findings [2]. Briefly justify the choice of the comparator.

	1	2	3	4	5	
subitem not at all important	○	○	○	○	◉	essential

清除選取

Does your paper address subitem 2a-ii? *

Copy and paste relevant sections from the manuscript (include quotes in quotation marks "like this" to indicate direct quotes from your manuscript), or elaborate on this item by providing additional information not in the ms, or briefly explain why the item is not applicable/relevant for your study

Tendon repair and suture techniques are part of the orthopedic course during clinical training. For traditional training, students only have limited time to practice due to cost limitations [15, 16]. During clinical practice, a real patient is involved at every step in the process. Because of the lack of practical training, medical students need to be guided through a long learning curve to become independent in clinical practice [17].

2b) In INTRODUCTION: Specific objectives or hypotheses

Does your paper address CONSORT subitem 2b? *

Copy and paste relevant sections from the manuscript (include quotes in quotation marks "like this" to indicate direct quotes from your manuscript), or elaborate on this item by providing additional information not in the ms, or briefly explain why the item is not applicable/relevant for your study

This study compared traditional tendon repair training and VR simulation of tendon repair and evaluated future applications of VR simulation in the academic medical field.

METHODS

3a) Description of trial design (such as parallel, factorial) including allocation ratio

Does your paper address CONSORT subitem 3a? *

Copy and paste relevant sections from the manuscript (include quotes in quotation marks "like this" to indicate direct quotes from your manuscript), or elaborate on this item by providing additional information not in the ms, or briefly explain why the item is not applicable/relevant for your study

This study is a parallel-design randomized controlled trial comparing VR and control groups. This study was approved by the ethics committee of the First Affiliated Hospital of Jinan University and registered in the Chinese Clinical Trial Registry (Reg No.: 2019-03-0262). Information was collected from all participants after obtaining written informed consent in accordance with the Declaration of Helsinki. All participants were required to complete the final assessment, which was performing tendon repair on synthetic models with 2 different knots: the “Kessler tendon suture with 2 interrupted tendon suture knots” and the “Bunnell tendon suture with figure 8 tendon suture” (KS and BS methods, respectively).

3b) Important changes to methods after trial commencement (such as eligibility criteria), with reasons

Does your paper address CONSORT subitem 3b? *

Copy and paste relevant sections from the manuscript (include quotes in quotation marks "like this" to indicate direct quotes from your manuscript), or elaborate on this item by providing additional information not in the ms, or briefly explain why the item is not applicable/relevant for your study

All participants provided written, informed, oral independently-witnessed consent to participate in the research study. A random allocation sequence was generated using a random number table. A sequence was used to allocate groups of participants to the VR and control groups. For the examination, the students performed tendon repair on a synthetic model. All participants were randomly assigned to one of the two groups. Participants in the VR group (n = 61) learned the technique of tendon repair through the VR simulator method, whereas the control group (n = 60) used the traditional tendon repair teaching method. The examiners were well-trained surgeons and unaffiliated with the medical school; they evaluated and assigned a score to each final product immediately without knowing the allocation list in a nonbiased manner during pre-and post-intervention assessments. In order to ensure the rigor of the examination, we have included a short training session for the examiners. Training helps to clarify the examiner's role, required behavior, review the marking guidance, marking assignment to standardize the exam, and encourage the consistency of the examiner's marking behavior. At the end of the training, examiners also did a marking exercise to scrutinize examiners' marking behavior. During the examination, medical students were asked not to tell the examiner which group they were assigned to.

3b-i) Bug fixes, Downtimes, Content Changes

Bug fixes, Downtimes, Content Changes: ehealth systems are often dynamic systems. A description of changes to methods therefore also includes important changes made on the intervention or comparator during the trial (e.g., major bug fixes or changes in the functionality or content) (5-iii) and other "unexpected events" that may have influenced study design such as staff changes, system failures/downtimes, etc. [2].

	1	2	3	4	5	
subitem not at all important	◉	○	○	○	○	essential

清除選取

Does your paper address subitem 3b-i?

Copy and paste relevant sections from the manuscript (include quotes in quotation marks "like this" to indicate direct quotes from your manuscript), or elaborate on this item by providing additional information not in the ms, or briefly explain why the item is not applicable/relevant for your study

No relevant issue with the Bug fixes, Downtimes, Content Changes.

4a) Eligibility criteria for participants

Does your paper address CONSORT subitem 4a? *

Copy and paste relevant sections from the manuscript (include quotes in quotation marks "like this" to indicate direct quotes from your manuscript), or elaborate on this item by providing additional information not in the ms, or briefly explain why the item is not applicable/relevant for your study

Senior medical students were eligible participants in this study. They were required to complete the following fundamental courses before entering the randomized control trial: (1) human anatomy, (2) physiology, (3) biochemistry, (4) pathology, (5) pathophysiology, (6) diagnostics, (7) internal medicine, (8) orthopedics, (9) surgical probation, and (10) other professional basic clinical courses. This study excluded any participant who did not meet the above requirement. Written informed consent with a clearly stated study plan was given to all participants. The purpose of this trial was explained to the participants. After informed consent had been signed, we asked the medical students to perform tendon repair on synthetic simulations. A baseline score was given by an orthopedic specialist. Other baseline information, including gender, age, and grade point average, was collected from the medical school database.

4a-i) Computer / Internet literacy

Computer / Internet literacy is often an implicit “de facto” eligibility criterion - this should be explicitly clarified.

	1	2	3	4	5	
subitem not at all important	○	○	○	○	◉	essential

清除選取

Does your paper address subitem 4a-i?

Copy and paste relevant sections from the manuscript (include quotes in quotation marks "like this" to indicate direct quotes from your manuscript), or elaborate on this item by providing additional information not in the ms, or briefly explain why the item is not applicable/relevant for your study

您的答案

4a-ii) Open vs. closed, web-based vs. face-to-face assessments:

Open vs. closed, web-based vs. face-to-face assessments: Mention how participants were recruited (online vs. offline), e.g., from an open access website or from a clinic, and clarify if this was a purely web-based trial, or there were face-to-face components (as part of the intervention or for assessment), i.e., to what degree got the study team to know the participant. In online-only trials, clarify if participants were quasi-anonymous and whether having multiple identities was possible or whether technical or logistical measures (e.g., cookies, email confirmation, phone calls) were used to detect/prevent these.

	1	2	3	4	5	
subitem not at all important	○	○	◉	○	○	essential

清除選取

Does your paper address subitem 4a-ii? *

Copy and paste relevant sections from the manuscript (include quotes in quotation marks "like this" to indicate direct quotes from your manuscript), or elaborate on this item by providing additional information not in the ms, or briefly explain why the item is not applicable/relevant for your study

The control group participants were required to participate in the full eight hours of lectures and a six-hour practical class in medical school for two weeks. The participants learned about traumatic orthopedic theory and the fundamentals of tendon repair during the lectures. They practiced tendon repair on the synthetic models under the professor’s guidance. In the practice class, students were given a PowerPoint, which provided illustrations, photographs, and step-by-step instructions. They were instructed to review the training material for one hour.

The VR group participants were required to take the same course as the control group, except the guided PowerPoint review part. Moreover, they practiced the VR simulators (including the VR version and the PC version) for one hour in class. The medical students practiced under the guidance with detailed instructions. The VR simulator is focused on every participant’s performance while performing tendon repair. The operation in the VR simulator is divided into practice and examination modes. Corresponding notes for each step during the practice mode were provided; however, no notes were provided for the examination mode (Appendix 1). The students were required to finish all the required learning in the practice mode before entering the examination mode for assessment. For the VR training section, while half of the students were practicing in the VR simulator, the rest of the students were practicing on the PC version in the training center. These students shifted the training modes after 30 minutes of VR training (see Table 1). All training was performed within the classes and both groups had exactly the same opportunity for practice time.

4a-iii) Information giving during recruitment

Information given during recruitment. Specify how participants were briefed for recruitment and in the informed consent procedures (e.g., publish the informed consent documentation as appendix, see also item X26), as this information may have an effect on user self-selection, user expectation and may also bias results.

	1	2	3	4	5	
subitem not at all important	○	○	○	○	◉	essential

清除選取

Does your paper address subitem 4a-iii?

Copy and paste relevant sections from the manuscript (include quotes in quotation marks "like this" to indicate direct quotes from your manuscript), or elaborate on this item by providing additional information not in the ms, or briefly explain why the item is not applicable/relevant for your study

All participants provided written, informed, oral independently-witnessed consent to participate in the research study. A random allocation sequence was generated using a random number table. A sequence was used to allocate groups of participants to the VR and control groups. For the examination, the students performed tendon repair on a synthetic model. All participants were randomly assigned to one of the two groups. Participants in the VR group (n = 61) learned the technique of tendon repair through the VR simulator method, whereas the control group (n = 60) used the traditional tendon repair teaching method. The examiners were well-trained surgeons and unaffiliated with the medical school; they evaluated and assigned a score to each final product immediately without knowing the allocation list in a nonbiased manner during pre-and post-intervention assessments. In order to ensure the rigor of the examination, we have included a short training session for the examiners. Training helps to clarify the examiner's role, required behavior, review the marking guidance, marking assignment to standardize the exam, and encourage the consistency of the examiner's marking behavior. At the end of the training, examiners also did a marking exercise to scrutinize examiners' marking behavior. During the examination, medical students were asked not to tell the examiner which group they were assigned to (Figure 1).

4b) Settings and locations where the data were collected

Does your paper address CONSORT subitem 4b? *

Copy and paste relevant sections from the manuscript (include quotes in quotation marks "like this" to indicate direct quotes from your manuscript), or elaborate on this item by providing additional information not in the ms, or briefly explain why the item is not applicable/relevant for your study

All participants provided written, informed, oral independently-witnessed consent to participate in the research study. A random allocation sequence was generated using a random number table. A sequence was used to allocate groups of participants to the VR and control groups. For the examination, the students performed tendon repair on a synthetic model. All participants were randomly assigned to one of the two groups. Participants in the VR group (n = 61) learned the technique of tendon repair through the VR simulator method, whereas the control group (n = 60) used the traditional tendon repair teaching method. The examiners were well-trained surgeons and unaffiliated with the medical school; they evaluated and assigned a score to each final product immediately without knowing the allocation list in a nonbiased manner during pre-and post-intervention assessments. In order to ensure the rigor of the examination, we have included a short training session for the examiners. Training helps to clarify the examiner's role, required behavior, review the marking guidance, marking assignment to standardize the exam, and encourage the consistency of the examiner's marking behavior. At the end of the training, examiners also did a marking exercise to scrutinize examiners' marking behavior. During the examination, medical students were asked not to tell the examiner which group they were assigned to (Figure 1).

4b-i) Report if outcomes were (self-)assessed through online questionnaires

Clearly report if outcomes were (self-)assessed through online questionnaires (as common in web-based trials) or otherwise.

	1	2	3	4	5	
subitem not at all important	(•)	( )	( )	( )	( )	essential

清除選取

Does your paper address subitem 4b-i? *

Copy and paste relevant sections from the manuscript (include quotes in quotation marks "like this" to indicate direct quotes from your manuscript), or elaborate on this item by providing additional information not in the ms, or briefly explain why the item is not applicable/relevant for your study

Both the control and experimental groups participated in the research study for 14 days. The results were calculated using the global rating scale (GRS). Seven dimensions were incorporated into the tool. The GRS shows different aspects of operative performance. This technology was compared using the global rating scale according to several aspects: (1) respect for tissue, (2) time in motion, (3) instrument handling, (4) tendon repair skill, (5) flow of operation, (6) knowledge of procedure and final suture, and (7) qualitative and objective assessment of all tendon repair performances [27, 28]. Each column is scored on a 5-point scale. Explicit descriptors were designed to guide the examiner when evaluating the students' performance. Each item is scored from 1 (poor performance) to 5 (good performance). Higher scores on the final assessment indicate that the participants using the specific method have better performance with respect to the tendon suturing technique on the total 35-point scale [27]. The GRS is widely used in the evaluation of surgical behavior, including objective and subjective criteria [29]. It has been used for various types of surgical operation evaluations, such as arthroscopic surgery [30], endoscopic surgery [31], pediatric surgery [32], and orthopedic surgery [33]. Additionally, it has been validated for use with VR systems because it measures nontechnical cognitive skills, such as decision-making and judgment [34].

4b-ii) Report how institutional affiliations are displayed

Report how institutional affiliations are displayed to potential participants [on ehealth media], as affiliations with prestigious hospitals or universities may affect volunteer rates, use, and reactions with regards to an intervention.(Not a required item – describe only if this may bias results)

	1	2	3	4	5	
subitem not at all important	○	○	○	○	◉	essential

清除選取

Does your paper address subitem 4b-ii?

Copy and paste relevant sections from the manuscript (include quotes in quotation marks "like this" to indicate direct quotes from your manuscript), or elaborate on this item by providing additional information not in the ms, or briefly explain why the item is not applicable/relevant for your study

The examiners were well-trained surgeons and unaffiliated with the medical school

5) The interventions for each group with sufficient details to allow replication, including how and when they were actually administered

5-i) Mention names, credential, affiliations of the developers, sponsors, and owners

Mention names, credential, affiliations of the developers, sponsors, and owners [6] (if authors/evaluators are owners or developer of the software, this needs to be declared in a “Conflict of interest” section or mentioned elsewhere in the manuscript).

	1	2	3	4	5	
subitem not at all important	○	○	○	○	◉	essential

清除選取

Does your paper address subitem 5-i?

Copy and paste relevant sections from the manuscript (include quotes in quotation marks "like this" to indicate direct quotes from your manuscript), or elaborate on this item by providing additional information not in the ms, or briefly explain why the item is not applicable/relevant for your study

The VR simulator used in this study was created by Jinan University and the Department of Orthopedic Surgery and Sports Medicine Center (http://www.ilab-x.com/details?id=2934&isView=true)[24]. All VR simulators were classified as HTC Vive VR[25], and the software was SteamVR (JinKe Lu, Shanghai, China)[26]. The VR simulator method used in this study included an independent-study section in which each participant was required to study all of the theories on the VR simulator website. This website is open to the public after registration on the website.

5-ii) Describe the history/development process

Describe the history/development process of the application and previous formative evaluations (e.g., focus groups, usability testing), as these will have an impact on adoption/use rates and help with interpreting results.

	1	2	3	4	5	
subitem not at all important	○	○	○	○	◉	essential

清除選取

Does your paper address subitem 5-ii?

Copy and paste relevant sections from the manuscript (include quotes in quotation marks "like this" to indicate direct quotes from your manuscript), or elaborate on this item by providing additional information not in the ms, or briefly explain why the item is not applicable/relevant for your study

None of the history/development process of the application and previous formative evaluations

5-iii) Revisions and updating

Revisions and updating. Clearly mention the date and/or version number of the application/intervention (and comparator, if applicable) evaluated, or describe whether the intervention underwent major changes during the evaluation process, or whether the development and/or content was "frozen" during the trial. Describe dynamic components such as news feeds or changing content which may have an impact on the replicability of the intervention (for unexpected events see item 3b).

	1	2	3	4	5	
subitem not at all important	○	○	○	○	◉	essential

清除選取

Does your paper address subitem 5-iii?

Copy and paste relevant sections from the manuscript (include quotes in quotation marks "like this" to indicate direct quotes from your manuscript), or elaborate on this item by providing additional information not in the ms, or briefly explain why the item is not applicable/relevant for your study

This is the first version of the application.

5-iv) Quality assurance methods

Provide information on quality assurance methods to ensure accuracy and quality of information provided [1], if applicable.

	1	2	3	4	5	
subitem not at all important	○	○	○	○	◉	essential

清除選取

Does your paper address subitem 5-iv?

Copy and paste relevant sections from the manuscript (include quotes in quotation marks "like this" to indicate direct quotes from your manuscript), or elaborate on this item by providing additional information not in the ms, or briefly explain why the item is not applicable/relevant for your study

The GRS is widely used in the evaluation of surgical behavior, including objective and subjective criteria [29]. It has been used for various types of surgical operation evaluations, such as arthroscopic surgery [30], endoscopic surgery [31], pediatric surgery [32], and orthopedic surgery [33]. Additionally, it has been validated for use with VR systems because it measures nontechnical cognitive skills, such as decision-making and judgment [34].

5-v) Ensure replicability by publishing the source code, and/or providing screenshots/screen-capture video, and/or providing flowcharts of the algorithms used

Ensure replicability by publishing the source code, and/or providing screenshots/screen-capture video, and/or providing flowcharts of the algorithms used. Replicability (i.e., other researchers should in principle be able to replicate the study) is a hallmark of scientific reporting.

	1	2	3	4	5	
subitem not at all important	○	○	○	○	◉	essential

清除選取

Does your paper address subitem 5-v?

Copy and paste relevant sections from the manuscript (include quotes in quotation marks "like this" to indicate direct quotes from your manuscript), or elaborate on this item by providing additional information not in the ms, or briefly explain why the item is not applicable/relevant for your study

Data were analyzed using the SPSS 23.0 (SPSS, Chicago, Illinois, USA) software package[35]. The baseline information, including age and GPA, was analyzed using the independent T test for parametric data [36]. Differences in the objective and semi-objective measurements between the two groups were analyzed using the Mann–Whitney analysis for nonparametric data [37]. The level of agreement between the semi-objective assessments made by the two experts was estimated by Cohen’s K coefficient. $P < 0.05$ was considered significant [38].

5-vi) Digital preservation

Digital preservation: Provide the URL of the application, but as the intervention is likely to change or disappear over the course of the years; also make sure the intervention is archived (Internet Archive, webcitation.org, and/or publishing the source code or screenshots/videos alongside the article). As pages behind login screens cannot be archived, consider creating demo pages which are accessible without login.

	1	2	3	4	5	
subitem not at all important	○	○	○	○	◉	essential

清除選取

Does your paper address subitem 5-vi?

Copy and paste relevant sections from the manuscript (include quotes in quotation marks "like this" to indicate direct quotes from your manuscript), or elaborate on this item by providing additional information not in the ms, or briefly explain why the item is not applicable/relevant for your study

http://www.ilab-x.com/details?id=2934&isView=true

5-vii) Access

Access: Describe how participants accessed the application, in what setting/context, if they had to pay (or were paid) or not, whether they had to be a member of specific group. If known, describe how participants obtained “access to the platform and Internet” [1]. To ensure access for editors/reviewers/readers, consider to provide a “backdoor” login account or demo mode for reviewers/readers to explore the application (also important for archiving purposes, see vi).

	1	2	3	4	5	
subitem not at all important	○	○	○	○	◉	essential

清除選取

Does your paper address subitem 5-vii? *

Copy and paste relevant sections from the manuscript (include quotes in quotation marks "like this" to indicate direct quotes from your manuscript), or elaborate on this item by providing additional information not in the ms, or briefly explain why the item is not applicable/relevant for your study

This website is open to the public after registration on the website.

5-viii) Mode of delivery, features/functionalities/components of the intervention and comparator, and the theoretical framework

Describe mode of delivery, features/functionalities/components of the intervention and comparator, and the theoretical framework [6] used to design them (instructional strategy [1], behaviour change techniques, persuasive features, etc., see e.g., [7, 8] for terminology). This includes an in-depth description of the content (including where it is coming from and who developed it) [1]," whether [and how] it is tailored to individual circumstances and allows users to track their progress and receive feedback" [6]. This also includes a description of communication delivery channels and – if computer-mediated communication is a component – whether communication was synchronous or asynchronous [6]. It also includes information on presentation strategies [1], including page design principles, average amount of text on pages, presence of hyperlinks to other resources, etc. [1].

	1	2	3	4	5	
subitem not at all important	○	○	○	○	◉	essential

清除選取

Does your paper address subitem 5-viii? *

Copy and paste relevant sections from the manuscript (include quotes in quotation marks "like this" to indicate direct quotes from your manuscript), or elaborate on this item by providing additional information not in the ms, or briefly explain why the item is not applicable/relevant for your study

Both versions were open to the students for practice according to their needs. In addition, the practical study section of tendon repair is performed using the VR simulator with individual steps for each procedure (comprising 7 steps in total). The website provides a section in which students and teachers can communicate and share ideas with each other. This program allows the students to learn in-depth while enhancing their learning through the communication section of the VR simulator. This online discussion can overcome the barriers of time and distance.

5-ix) Describe use parameters

Describe use parameters (e.g., intended "doses" and optimal timing for use). Clarify what instructions or recommendations were given to the user, e.g., regarding timing, frequency, heaviness of use, if any, or was the intervention used ad libitum.

	1	2	3	4	5	
subitem not at all important	○	○	○	○	◉	essential

清除選取

Does your paper address subitem 5-ix?

Copy and paste relevant sections from the manuscript (include quotes in quotation marks "like this" to indicate direct quotes from your manuscript), or elaborate on this item by providing additional information not in the ms, or briefly explain why the item is not applicable/relevant for your study

Both the control and experimental groups participated in the research study for 14 days.

5-x) Clarify the level of human involvement

Clarify the level of human involvement (care providers or health professionals, also technical assistance) in the e-intervention or as co-intervention (detail number and expertise of professionals involved, if any, as well as "type of assistance offered, the timing and frequency of the support, how it is initiated, and the medium by which the assistance is delivered". It may be necessary to distinguish between the level of human involvement required for the trial, and the level of human involvement required for a routine application outside of a RCT setting (discuss under item 21 – generalizability).

	1	2	3	4	5	
subitem not at all important	○	○	○	○	◉	essential

清除選取

Does your paper address subitem 5-x?

Copy and paste relevant sections from the manuscript (include quotes in quotation marks "like this" to indicate direct quotes from your manuscript), or elaborate on this item by providing additional information not in the ms, or briefly explain why the item is not applicable/relevant for your study

This program allows the students to learn in-depth while enhancing their learning through the communication section of the VR simulator.

5-xi) Report any prompts/reminders used

Report any prompts/reminders used: Clarify if there were prompts (letters, emails, phone calls, SMS) to use the application, what triggered them, frequency etc. It may be necessary to distinguish between the level of prompts/reminders required for the trial, and the level of prompts/reminders for a routine application outside of a RCT setting (discuss under item 21 – generalizability).

	1	2	3	4	5	
subitem not at all important	◉	○	○	○	○	essential

清除選取

Does your paper address subitem 5-xi? *

Copy and paste relevant sections from the manuscript (include quotes in quotation marks "like this" to indicate direct quotes from your manuscript), or elaborate on this item by providing additional information not in the ms, or briefly explain why the item is not applicable/relevant for your study

No prompts (letters, emails, phone calls, SMS) to use the application was applied.

5-xii) Describe any co-interventions (incl. training/support)

Describe any co-interventions (incl. training/support): Clearly state any interventions that are provided in addition to the targeted eHealth intervention, as ehealth intervention may not be designed as stand-alone intervention. This includes training sessions and support [1]. It may be necessary to distinguish between the level of training required for the trial, and the level of training for a routine application outside of a RCT setting (discuss under item 21 – generalizability.

	1	2	3	4	5	
subitem not at all important	○	○	○	○	◉	essential

清除選取

Does your paper address subitem 5-xii? *

Copy and paste relevant sections from the manuscript (include quotes in quotation marks "like this" to indicate direct quotes from your manuscript), or elaborate on this item by providing additional information not in the ms, or briefly explain why the item is not applicable/relevant for your study

The VR group participants were required to take the same course as the control group, except the guided PowerPoint review part. Moreover, they practiced the VR simulators (including the VR version and the PC version) for one hour in class.

6a) Completely defined pre-specified primary and secondary outcome measures, including how and when they were assessed

Does your paper address CONSORT subitem 6a? *

Copy and paste relevant sections from the manuscript (include quotes in quotation marks "like this" to indicate direct quotes from your manuscript), or elaborate on this item by providing additional information not in the ms, or briefly explain why the item is not applicable/relevant for your study

This study was approved by the ethics committee of the First Affiliated Hospital of Jinan University and registered in the Chinese Clinical Trial Registry (Reg No.: 2019-03-0262). Information was collected from all participants after obtaining written informed consent in accordance with the Declaration of Helsinki. All participants were required to complete the final assessment, which was performing tendon repair on synthetic models with 2 different knots: the "Kessler tendon suture with 2 interrupted tendon suture knots" and the "Bunnell tendon suture with figure 8 tendon suture" (KS and BS methods, respectively).

6a-i) Online questionnaires: describe if they were validated for online use and apply CHERRIES items to describe how the questionnaires were designed/deployed

If outcomes were obtained through online questionnaires, describe if they were validated for online use and apply CHERRIES items to describe how the questionnaires were designed/deployed [9].

	1	2	3	4	5	
subitem not at all important	○	○	○	○	◉	essential

清除選取

Does your paper address subitem 6a-i?

Copy and paste relevant sections from manuscript text

No online questionnaires were used.

6a-ii) Describe whether and how “use” (including intensity of use/dosage) was defined/measured/monitored

Describe whether and how “use” (including intensity of use/dosage) was defined/measured/monitored (logins, logfile analysis, etc.). Use/adoption metrics are important process outcomes that should be reported in any ehealth trial.

	1	2	3	4	5	
subitem not at all important	○	○	○	○	◉	essential

清除選取

Does your paper address subitem 6a-ii?

Copy and paste relevant sections from manuscript text

No usage was measured in this trial.

6a-iii) Describe whether, how, and when qualitative feedback from participants was obtained

Describe whether, how, and when qualitative feedback from participants was obtained (e.g., through emails, feedback forms, interviews, focus groups).

	1	2	3	4	5	
subitem not at all important	○	○	○	○	◉	essential

清除選取

Does your paper address subitem 6a-iii?

Copy and paste relevant sections from manuscript text

qualitative feedback from participants was not obtained

6b) Any changes to trial outcomes after the trial commenced, with reasons

Does your paper address CONSORT subitem 6b? *

Copy and paste relevant sections from the manuscript (include quotes in quotation marks "like this" to indicate direct quotes from your manuscript), or elaborate on this item by providing additional information not in the ms, or briefly explain why the item is not applicable/relevant for your study

No changes to trial outcomes after the trial commenced

7a) How sample size was determined

NPT: When applicable, details of whether and how the clustering by care provides or centers was addressed

7a-i) Describe whether and how expected attrition was taken into account when calculating the sample size

Describe whether and how expected attrition was taken into account when calculating the sample size.

	1	2	3	4	5	
subitem not at all important	○	○	○	○	◉	essential

清除選取

Does your paper address subitem 7a-i?

Copy and paste relevant sections from manuscript title (include quotes in quotation marks "like this" to indicate direct quotes from your manuscript), or elaborate on this item by providing additional information not in the ms, or briefly explain why the item is not applicable/relevant for your study

Participants in the VR group (n = 61) learned the technique of tendon repair through the VR simulator method, whereas the control group (n = 60) used the traditional tendon repair teaching method.

7b) When applicable, explanation of any interim analyses and stopping guidelines

Does your paper address CONSORT subitem 7b? *

Copy and paste relevant sections from the manuscript (include quotes in quotation marks "like this" to indicate direct quotes from your manuscript), or elaborate on this item by providing additional information not in the ms, or briefly explain why the item is not applicable/relevant for your study

No any interim analyses and stopping guidelines.

8a) Method used to generate the random allocation sequence

NPT: When applicable, how care providers were allocated to each trial group

Does your paper address CONSORT subitem 8a? *

Copy and paste relevant sections from the manuscript (include quotes in quotation marks "like this" to indicate direct quotes from your manuscript), or elaborate on this item by providing additional information not in the ms, or briefly explain why the item is not applicable/relevant for your study

A random allocation sequence was generated using a random number table. A sequence was used to allocate groups of participants to the VR and control groups.

8b) Type of randomisation; details of any restriction (such as blocking and block size)

Does your paper address CONSORT subitem 8b? *

Copy and paste relevant sections from the manuscript (include quotes in quotation marks "like this" to indicate direct quotes from your manuscript), or elaborate on this item by providing additional information not in the ms, or briefly explain why the item is not applicable/relevant for your study

This study is a parallel-design randomized controlled trial comparing VR and control groups.

9) Mechanism used to implement the random allocation sequence (such as sequentially numbered containers), describing any steps taken to conceal the sequence until interventions were assigned

Does your paper address CONSORT subitem 9? *

Copy and paste relevant sections from the manuscript (include quotes in quotation marks "like this" to indicate direct quotes from your manuscript), or elaborate on this item by providing additional information not in the ms, or briefly explain why the item is not applicable/relevant for your study

A random allocation sequence was generated using a random number table. A sequence was used to allocate groups of participants to the VR and control groups.

10) Who generated the random allocation sequence, who enrolled participants, and who assigned participants to interventions

Does your paper address CONSORT subitem 10? *

Copy and paste relevant sections from the manuscript (include quotes in quotation marks "like this" to indicate direct quotes from your manuscript), or elaborate on this item by providing additional information not in the ms, or briefly explain why the item is not applicable/relevant for your study

A random allocation sequence was generated using a random number table. A sequence was used to allocate groups of participants to the VR and control groups.

11a) If done, who was blinded after assignment to interventions (for example, participants, care providers, those assessing outcomes) and how

NPT: Whether or not administering co-interventions were blinded to group assignment

11a-i) Specify who was blinded, and who wasn't

Specify who was blinded, and who wasn't. Usually, in web-based trials it is not possible to blind the participants [1, 3] (this should be clearly acknowledged), but it may be possible to blind outcome assessors, those doing data analysis or those administering co-interventions (if any).

	1	2	3	4	5	
subitem not at all important	○	○	○	○	◉	essential

清除選取

Does your paper address subitem 11a-i? *

Copy and paste relevant sections from the manuscript (include quotes in quotation marks "like this" to indicate direct quotes from your manuscript), or elaborate on this item by providing additional information not in the ms, or briefly explain why the item is not applicable/relevant for your study

At the end of the training, examiners also did a marking exercise to scrutinize examiners' marking behavior. During the examination, medical students were asked not to tell the examiner which group they were assigned to (Figure 1).

11a-ii) Discuss e.g., whether participants knew which intervention was the “intervention of interest” and which one was the “comparator”

Informed consent procedures (4a-ii) can create biases and certain expectations - discuss e.g., whether participants knew which intervention was the “intervention of interest” and which one was the “comparator”.

	1	2	3	4	5	
subitem not at all important	○	○	○	○	◉	essential

清除選取

Does your paper address subitem 11a-ii?

Copy and paste relevant sections from the manuscript (include quotes in quotation marks "like this" to indicate direct quotes from your manuscript), or elaborate on this item by providing additional information not in the ms, or briefly explain why the item is not applicable/relevant for your study

All participants provided written, informed, oral independently-witnessed consent to participate in the research study.

11b) If relevant, description of the similarity of interventions

(this item is usually not relevant for ehealth trials as it refers to similarity of a placebo or sham intervention to a active medication/intervention)

Does your paper address CONSORT subitem 11b? *

Copy and paste relevant sections from the manuscript (include quotes in quotation marks "like this" to indicate direct quotes from your manuscript), or elaborate on this item by providing additional information not in the ms, or briefly explain why the item is not applicable/relevant for your study

The control group participants were required to participate in the full eight hours of lectures and a six-hour practical class in medical school for two weeks. The participants learned about traumatic orthopedic theory and the fundamentals of tendon repair during the lectures. They practiced tendon repair on the synthetic models under the professor's guidance. In the practice class, students were given a PowerPoint, which provided illustrations, photographs, and step-by-step instructions. They were instructed to review the training material for one hour.

The VR group participants were required to take the same course as the control group, except the guided PowerPoint review part. Moreover, they practiced the VR simulators (including the VR version and the PC version) for one hour in class. The medical students practiced under the guidance with detailed instructions. The VR simulator is focused on every participant's performance while performing tendon repair. The operation in the VR simulator is divided into practice and examination modes. Corresponding notes for each step during the practice mode were provided; however, no notes were provided for the examination mode (Appendix 1). The students were required to finish all the required learning in the practice mode before entering the examination mode for assessment. For the VR training section, while half of the students were practicing in the VR simulator, the rest of the students were practicing on the PC version in the training center. These students shifted the training modes after 30 minutes of VR training (see Table 1). All training was performed within the classes and both groups had exactly the same opportunity for practice time.

12a) Statistical methods used to compare groups for primary and secondary outcomes

NPT: When applicable, details of whether and how the clustering by care providers or centers was addressed

Does your paper address CONSORT subitem 12a? *

Copy and paste relevant sections from the manuscript (include quotes in quotation marks "like this" to indicate direct quotes from your manuscript), or elaborate on this item by providing additional information not in the ms, or briefly explain why the item is not applicable/relevant for your study

Data were analyzed using the SPSS 23.0 (SPSS, Chicago, Illinois, USA) software package[35]. The baseline information, including age and GPA, was analyzed using the independent T test for parametric data [36]. Differences in the objective and semi-objective measurements between the two groups were analyzed using the Mann–Whitney analysis for nonparametric data [37]. The level of agreement between the semi-objective assessments made by the two experts was estimated by Cohen’s K coefficient. $P < 0.05$ was considered significant [38].

12a-i) Imputation techniques to deal with attrition / missing values

Imputation techniques to deal with attrition / missing values: Not all participants will use the intervention/comparator as intended and attrition is typically high in ehealth trials. Specify how participants who did not use the application or dropped out from the trial were treated in the statistical analysis (a complete case analysis is strongly discouraged, and simple imputation techniques such as LOCF may also be problematic [4]).

	1	2	3	4	5	
subitem not at all important	○	○	○	○	◉	essential

清除選取

Does your paper address subitem 12a-i? *

Copy and paste relevant sections from the manuscript (include quotes in quotation marks "like this" to indicate direct quotes from your manuscript), or elaborate on this item by providing additional information not in the ms, or briefly explain why the item is not applicable/relevant for your study

Imputation techniques to deal with attrition / missing values:

12b) Methods for additional analyses, such as subgroup analyses and adjusted analyses

Does your paper address CONSORT subitem 12b? *

Copy and paste relevant sections from the manuscript (include quotes in quotation marks "like this" to indicate direct quotes from your manuscript), or elaborate on this item by providing additional information not in the ms, or briefly explain why the item is not applicable/relevant for your study

The level of agreement between the semi-objective assessments made by the two experts was estimated by Cohen's K coefficient. $P < 0.05$ was considered significant [38].

X26) REB/IRB Approval and Ethical Considerations [recommended as subheading under "Methods"] (not a CONSORT item)

X26-i) Comment on ethics committee approval

	1	2	3	4	5	
subitem not at all important	○	○	○	○	◉	essential

清除選取

Does your paper address subitem X26-i?

Copy and paste relevant sections from the manuscript (include quotes in quotation marks "like this" to indicate direct quotes from your manuscript), or elaborate on this item by providing additional information not in the ms, or briefly explain why the item is not applicable/relevant for your study

This study was approved by the ethics committee of the First Affiliated Hospital of Jinan University and registered in the Chinese Clinical Trial Registry (Reg No.: 2019-03-0262). Information was collected from all participants after obtaining written informed consent in accordance with the Declaration of Helsinki.

x26-ii) Outline informed consent procedures

Outline informed consent procedures e.g., if consent was obtained offline or online (how? Checkbox, etc.?), and what information was provided (see 4a-ii). See [6] for some items to be included in informed consent documents.

	1	2	3	4	5	
subitem not at all important	○	○	○	○	◉	essential

清除選取

Does your paper address subitem X26-ii?

Copy and paste relevant sections from the manuscript (include quotes in quotation marks "like this" to indicate direct quotes from your manuscript), or elaborate on this item by providing additional information not in the ms, or briefly explain why the item is not applicable/relevant for your study

All participants provided written, informed, oral independently-witnessed consent to participate in the research study.

X26-iii) Safety and security procedures

Safety and security procedures, incl. privacy considerations, and any steps taken to reduce the likelihood or detection of harm (e.g., education and training, availability of a hotline)

	1	2	3	4	5	
subitem not at all important	○	○	○	○	◉	essential

清除選取

Does your paper address subitem X26-iii?

Copy and paste relevant sections from the manuscript (include quotes in quotation marks "like this" to indicate direct quotes from your manuscript), or elaborate on this item by providing additional information not in the ms, or briefly explain why the item is not applicable/relevant for your study

All personal information was blocked from the trial registry.

RESULTS

13a) For each group, the numbers of participants who were randomly assigned, received intended treatment, and were analysed for the primary outcome

NPT: The number of care providers or centers performing the intervention in each group and the number of patients treated by each care provider in each center

Does your paper address CONSORT subitem 13a? *

Copy and paste relevant sections from the manuscript (include quotes in quotation marks "like this" to indicate direct quotes from your manuscript), or elaborate on this item by providing additional information not in the ms, or briefly explain why the item is not applicable/relevant for your study

Between August 1, 2019, and August 12, 2020, 121 potential participants were assessed for study participation in the Medical School of Jinan University. Four participants from the control group dropped out of the program for personal reasons. All participants were required to undergo a final assessment on synthetic models, and the overall score sheet was used to calculate the results. This study analyzed all participants using the assessment global rating scale described above. The global rating scale baseline is shown for assessing tendon repair differences in the control and VR groups (Table 2). A comparison of participants in both groups according to age, gender, grade point average (GPA), and pretest evaluation revealed no educationally relevant or significant differences. Follow-up ended on September 30, 2020.

13b) For each group, losses and exclusions after randomisation, together with reasons

Does your paper address CONSORT subitem 13b? (NOTE: Preferably, this is shown in a CONSORT flow diagram) *

Copy and paste relevant sections from the manuscript (include quotes in quotation marks "like this" to indicate direct quotes from your manuscript), or elaborate on this item by providing additional information not in the ms, or briefly explain why the item is not applicable/relevant for your study

Between August 1, 2019, and August 12, 2020, 121 potential participants were assessed for study participation in the Medical School of Jinan University. Four participants from the control group dropped out of the program for personal reasons. All participants were required to undergo a final assessment on synthetic models, and the overall score sheet was used to calculate the results. This study analyzed all participants using the assessment global rating scale described above. The global rating scale baseline is shown for assessing tendon repair differences in the control and VR groups (Table 2). A comparison of participants in both groups according to age, gender, grade point average (GPA), and pretest evaluation revealed no educationally relevant or significant differences. Follow-up ended on September 30, 2020.

13b-i) Attrition diagram

Strongly recommended: An attrition diagram (e.g., proportion of participants still logging in or using the intervention/comparator in each group plotted over time, similar to a survival curve) or other figures or tables demonstrating usage/dose/engagement.

	1	2	3	4	5	
subitem not at all important	○	○	○	○	◉	essential

清除選取

Does your paper address subitem 13b-i?

Copy and paste relevant sections from the manuscript or cite the figure number if applicable (include quotes in quotation marks "like this" to indicate direct quotes from your manuscript), or elaborate on this item by providing additional information not in the ms, or briefly explain why the item is not applicable/relevant for your study

Posttraining on the global rating scale was used to assess tendon repair in the two groups. The table shows a comparison between the KS and BS methods. With respect to tissue, no significant difference using the KS method was found (p= 0.215). The control and VR groups were not significantly different using the BS method (p = 0.209) (Table 3).

14a) Dates defining the periods of recruitment and follow-up

Does your paper address CONSORT subitem 14a? *

Copy and paste relevant sections from the manuscript (include quotes in quotation marks "like this" to indicate direct quotes from your manuscript), or elaborate on this item by providing additional information not in the ms, or briefly explain why the item is not applicable/relevant for your study

Between August 1, 2019, and August 12, 2020, 121 potential participants were assessed for study participation in the Medical School of Jinan University. Four participants from the control group dropped out of the program for personal reasons. All participants were required to undergo a final assessment on synthetic models, and the overall score sheet was used to calculate the results. This study analyzed all participants using the assessment global rating scale described above. The global rating scale baseline is shown for assessing tendon repair differences in the control and VR groups (Table 2). A comparison of participants in both groups according to age, gender, grade point average (GPA), and pretest evaluation revealed no educationally relevant or significant differences. Follow-up ended on September 30, 2020.

14a-i) Indicate if critical “secular events” fell into the study period

Indicate if critical “secular events” fell into the study period, e.g., significant changes in Internet resources available or “changes in computer hardware or Internet delivery resources”

	1	2	3	4	5	
subitem not at all important	○	○	○	○	◉	essential

清除選取

Does your paper address subitem 14a-i?

Copy and paste relevant sections from the manuscript (include quotes in quotation marks "like this" to indicate direct quotes from your manuscript), or elaborate on this item by providing additional information not in the ms, or briefly explain why the item is not applicable/relevant for your study

No significant changes in Internet resources available or “changes in computer hardware or Internet delivery resources”

14b) Why the trial ended or was stopped (early)

Does your paper address CONSORT subitem 14b? *

Copy and paste relevant sections from the manuscript (include quotes in quotation marks "like this" to indicate direct quotes from your manuscript), or elaborate on this item by providing additional information not in the ms, or briefly explain why the item is not applicable/relevant for your study

Between August 1, 2019, and August 12, 2020, 121 potential participants were assessed for study participation in the Medical School of Jinan University. Four participants from the control group dropped out of the program for personal reasons. All participants were required to undergo a final assessment on synthetic models, and the overall score sheet was used to calculate the results. This study analyzed all participants using the assessment global rating scale described above. The global rating scale baseline is shown for assessing tendon repair differences in the control and VR groups (Table 2). A comparison of participants in both groups according to age, gender, grade point average (GPA), and pretest evaluation revealed no educationally relevant or significant differences. Follow-up ended on September 30, 2020.

15) A table showing baseline demographic and clinical characteristics for each group

NPT: When applicable, a description of care providers (case volume, qualification, expertise, etc.) and centers (volume) in each group

Does your paper address CONSORT subitem 15? *

Copy and paste relevant sections from the manuscript (include quotes in quotation marks "like this" to indicate direct quotes from your manuscript), or elaborate on this item by providing additional information not in the ms, or briefly explain why the item is not applicable/relevant for your study

Table 2: Baseline characteristics
Control Group (n=57) VR Group (n=61) P-value
Age
Gender in Male (%)
Grade Point Average 23.07 (0.97)
24 (42.9%)
3.02 (0.54) 22.93 (1.01)
31 (50.8%)
3.19 (0.49) 0.458
0.393
0.660
Kessler suture with 2 interrupted suture knots 8.00(7-9) 8.00(7-9) 0.132
Bunnell suture with figure-eight suture knots 8.00(7-9) 8.00(7-9) 0.253
Data are number of medical students (%), median (IQR), or mean (SD).

15-i) Report demographics associated with digital divide issues

In ehealth trials it is particularly important to report demographics associated with digital divide issues, such as age, education, gender, social-economic status, computer/Internet/ehealth literacy of the participants, if known.

	1	2	3	4	5	
subitem not at all important	○	○	○	○	◉	essential

清除選取

Does your paper address subitem 15-i? *

Copy and paste relevant sections from the manuscript (include quotes in quotation marks "like this" to indicate direct quotes from your manuscript), or elaborate on this item by providing additional information not in the ms, or briefly explain why the item is not applicable/relevant for your study

Table 2: Baseline characteristics
Control Group (n=57) VR Group (n=61) P-value
Age
Gender in Male (%)
Grade Point Average 23.07 (0.97)
24 (42.9%)
3.02 (0.54) 22.93 (1.01)
31 (50.8%)
3.19 (0.49) 0.458
0.393
0.660
Kessler suture with 2 interrupted suture knots 8.00(7-9) 8.00(7-9) 0.132
Bunnell suture with figure-eight suture knots 8.00(7-9) 8.00(7-9) 0.253
Data are number of medical students (%), median (IQR), or mean (SD).

16) For each group, number of participants (denominator) included in each analysis and whether the analysis was by original assigned groups

16-i) Report multiple "denominators" and provide definitions

Report multiple "denominators" and provide definitions: Report N's (and effect sizes) "across a range of study participation [and use] thresholds" [1], e.g., N exposed, N consented, N used more than x times, N used more than y weeks, N participants "used" the intervention/comparator at specific pre-defined time points of interest (in absolute and relative numbers per group). Always clearly define "use" of the intervention.

	1	2	3	4	5	
subitem not at all important	○	○	○	○	◉	essential

清除選取

Does your paper address subitem 16-i? *

Copy and paste relevant sections from the manuscript (include quotes in quotation marks "like this" to indicate direct quotes from your manuscript), or elaborate on this item by providing additional information not in the ms, or briefly explain why the item is not applicable/relevant for your study

No report multiple “denominators” and provide definitions

16-ii) Primary analysis should be intent-to-treat

Primary analysis should be intent-to-treat, secondary analyses could include comparing only “users”, with the appropriate caveats that this is no longer a randomized sample (see 18-i).

	1	2	3	4	5	
subitem not at all important	○	○	○	○	◉	essential

清除選取

Does your paper address subitem 16-ii?

Copy and paste relevant sections from the manuscript (include quotes in quotation marks "like this" to indicate direct quotes from your manuscript), or elaborate on this item by providing additional information not in the ms, or briefly explain why the item is not applicable/relevant for your study

The overall performance under the KS method was significant ($p < 0.001$) after comparing the control and VR groups. Regarding overall performance, the VR group performed better than the control group. The VR group under the BS method also showed a significantly better result than the control group ($p < 0.01$).

17a) For each primary and secondary outcome, results for each group, and the estimated effect size and its precision (such as 95% confidence interval)

Does your paper address CONSORT subitem 17a? *

Copy and paste relevant sections from the manuscript (include quotes in quotation marks "like this" to indicate direct quotes from your manuscript), or elaborate on this item by providing additional information not in the ms, or briefly explain why the item is not applicable/relevant for your study

Global rating scale Control Group (n=57) VR Group (n=61) p-value
Bunnell suture with figure-eight suture
Respect for tissue
1
2
3
4
5
0 (0.0%)
15 (26.8%)
41 (73.2%)
0 (0.0%)
0 (0.0%)
0 (0.0%)
23 (37.7%)
38 (62.3%)
0 (0.0%)
0 (0.0%) 0.209
Time in motion
1
2
3
4
5
0 (0.0%)
15 (26.8%)
41 (73.2%)
0 (0.0%)
0 (0.0%)
0 (0.0%)
5 (8.2%)
32 (52.5%)
23 (73.7%)
1 (1.6%) <0.001
Instrument handling
1
2
3
4
5
0 (0.0%)
8 (14.3%)
32 (57.1%)
16 (28.6%)
0 (0.0%)
0 (0.0%)

15 (24.6%)
33 (54.1%)
13 (21.3%)
0 (0.0%) 0.156
Suture skill
1
2
3
4
5
0 (0.0%)
17 (30.4%)
39 (69.6%)
0 (0.0%)
0 (0.0%)
0 (0.0%)
10 (16.4%)
30 (49.2%)
21 (34.1%)
0 (0.0%) <0.001
Flow of operation
1
2
3
4
5
0 (0.0%)
27 (48.2%)
25 (44.6%)
0 (0.0%)
4 (7.1%)
0 (0.0%)
1 (1.6%)
26 (42.6%)
28 (45.9%)
6 (9.8%) <0.001
Knowledge of procedure
1
2
3
4
5
0 (0.0%)
21 (37.5%)
21 (37.5%)
14 (25.0%)
0 (0.0%)
0 (0.0%)
2 (3.3%)
11 (18.0%)
47 (77.0%)
1 (1.6%) <0.001

Final product
1
2
3
4
5
0 (0.0%)
8 (14.3%)
41 (73.2%)
1 (1.8%)
6 (10.7%)
0 (0.0%)
11 (18.0%)
31 (50.8%)
9 (14.8%)
10 (16.4%) <0.001
Overall performance 20.00 (16-23) 23.00 (20-26) <0.001

17a-i) Presentation of process outcomes such as metrics of use and intensity of use

In addition to primary/secondary (clinical) outcomes, the presentation of process outcomes such as metrics of use and intensity of use (dose, exposure) and their operational definitions is critical. This does not only refer to metrics of attrition (13-b) (often a binary variable), but also to more continuous exposure metrics such as “average session length”. These must be accompanied by a technical description how a metric like a “session” is defined (e.g., timeout after idle time) [1] (report under item 6a).

	1	2	3	4	5	
subitem not at all important	○	○	○	○	◉	essential

清除選取

Does your paper address subitem 17a-i?

Copy and paste relevant sections from the manuscript (include quotes in quotation marks "like this" to indicate direct quotes from your manuscript), or elaborate on this item by providing additional information not in the ms, or briefly explain why the item is not applicable/relevant for your study

The overall performance under the KS method was significant ($p < 0.001$) after comparing the control and VR groups. Regarding overall performance, the VR group performed better than the control group. The VR group under the BS method also showed a significantly better result than the control group ($p < 0.01$).

17b) For binary outcomes, presentation of both absolute and relative effect sizes is recommended

Does your paper address CONSORT subitem 17b? *

Copy and paste relevant sections from the manuscript (include quotes in quotation marks "like this" to indicate direct quotes from your manuscript), or elaborate on this item by providing additional information not in the ms, or briefly explain why the item is not applicable/relevant for your study

Global rating scale Control Group (n=57) VR Group (n=61) p-value
Bunnell suture with figure-eight suture
Respect for tissue
1
2
3
4
5
0 (0.0%)
15 (26.8%)
41 (73.2%)
0 (0.0%)
0 (0.0%)
0 (0.0%)
23 (37.7%)
38 (62.3%)
0 (0.0%)
0 (0.0%) 0.209
Time in motion
1
2
3
4
5
0 (0.0%)
15 (26.8%)
41 (73.2%)
0 (0.0%)
0 (0.0%)
0 (0.0%)
5 (8.2%)
32 (52.5%)
23 (73.7%)
1 (1.6%) <0.001
Instrument handling
1
2
3
4
5
0 (0.0%)
8 (14.3%)
32 (57.1%)
16 (28.6%)
0 (0.0%)
0 (0.0%)

[illegible]
15 (24.6%)
33 (54.1%)
13 (21.3%)
0 (0.0%) 0.156
Suture skill
1
2
3
4
5
0 (0.0%)
17 (30.4%)
39 (69.6%)
0 (0.0%)
0 (0.0%)
0 (0.0%)
10 (16.4%)
30 (49.2%)
21 (34.1%)
0 (0.0%) <0.001
Flow of operation
1
2
3
4
5
0 (0.0%)
27 (48.2%)
25 (44.6%)
0 (0.0%)
4 (7.1%)
0 (0.0%)
1 (1.6%)
26 (42.6%)
28 (45.9%)
6 (9.8%) <0.001
Knowledge of procedure
1
2
3
4
5
0 (0.0%)
21 (37.5%)
21 (37.5%)
14 (25.0%)
0 (0.0%)
0 (0.0%)
2 (3.3%)
11 (18.0%)
47 (77.0%)
1 (1.6%) <0.001

Final product
1
2
3
4
5
0 (0.0%)
8 (14.3%)
41 (73.2%)
1 (1.8%)
6 (10.7%)
0 (0.0%)
11 (18.0%)
31 (50.8%)
9 (14.8%)
10 (16.4%) <0.001
Overall performance 20.00 (16-23) 23.00 (20-26) <0.001

18) Results of any other analyses performed, including subgroup analyses and adjusted analyses, distinguishing pre-specified from exploratory

Does your paper address CONSORT subitem 18? *

Copy and paste relevant sections from the manuscript (include quotes in quotation marks "like this" to indicate direct quotes from your manuscript), or elaborate on this item by providing additional information not in the ms, or briefly explain why the item is not applicable/relevant for your study

No other analyses performed, including subgroup analyses and adjusted analyses, distinguishing pre-specified from exploratory

18-i) Subgroup analysis of comparing only users

A subgroup analysis of comparing only users is not uncommon in ehealth trials, but if done, it must be stressed that this is a self-selected sample and no longer an unbiased sample from a randomized trial (see 16-iii).

	1	2	3	4	5	
subitem not at all important	○	○	○	○	◉	essential

清除選取

Does your paper address subitem 18-i?

Copy and paste relevant sections from the manuscript (include quotes in quotation marks "like this" to indicate direct quotes from your manuscript), or elaborate on this item by providing additional information not in the ms, or briefly explain why the item is not applicable/relevant for your study

您的答案

19) All important harms or unintended effects in each group

(for specific guidance see CONSORT for harms)

Does your paper address CONSORT subitem 19? *

Copy and paste relevant sections from the manuscript (include quotes in quotation marks "like this" to indicate direct quotes from your manuscript), or elaborate on this item by providing additional information not in the ms, or briefly explain why the item is not applicable/relevant for your study

No important harms or unintended effects in each group.

19-i) Include privacy breaches, technical problems

Include privacy breaches, technical problems. This does not only include physical “harm” to participants, but also incidents such as perceived or real privacy breaches [1], technical problems, and other unexpected/unintended incidents. “Unintended effects” also includes unintended positive effects [2].

	1	2	3	4	5	
subitem not at all important	○	○	○	○	◉	essential

清除選取

Does your paper address subitem 19-i?

Copy and paste relevant sections from the manuscript (include quotes in quotation marks "like this" to indicate direct quotes from your manuscript), or elaborate on this item by providing additional information not in the ms, or briefly explain why the item is not applicable/relevant for your study

This does not only include physical “harm” to participants, but also incidents such as perceived or real privacy breaches.

19-ii) Include qualitative feedback from participants or observations from staff/researchers

Include qualitative feedback from participants or observations from staff/researchers, if available, on strengths and shortcomings of the application, especially if they point to unintended/unexpected effects or uses. This includes (if available) reasons for why people did or did not use the application as intended by the developers.

	1	2	3	4	5	
subitem not at all important	○	○	○	○	◉	essential

清除選取

Does your paper address subitem 19-ii?

Copy and paste relevant sections from the manuscript (include quotes in quotation marks "like this" to indicate direct quotes from your manuscript), or elaborate on this item by providing additional information not in the ms, or briefly explain why the item is not applicable/relevant for your study

GRS is a semi-objective scale.

DISCUSSION

22) Interpretation consistent with results, balancing benefits and harms, and considering other relevant evidence

NPT: In addition, take into account the choice of the comparator, lack of or partial blinding, and unequal expertise of care providers or centers in each group

22-i) Restate study questions and summarize the answers suggested by the data, starting with primary outcomes and process outcomes (use)

Restate study questions and summarize the answers suggested by the data, starting with primary outcomes and process outcomes (use).

	1	2	3	4	5	
subitem not at all important	○	○	○	○	◉	essential

清除選取

Does your paper address subitem 22-i? *

Copy and paste relevant sections from the manuscript (include quotes in quotation marks "like this" to indicate direct quotes from your manuscript), or elaborate on this item by providing additional information not in the ms, or briefly explain why the item is not applicable/relevant for your study

In the field of orthopedics, researchers mainly focus on sophisticated surgical procedures, for example, (thoracic) pedicle screw placement and insertion (lateral mass screw placement) [42-44], (percutaneous) vertebroplasty [45], knee arthroscopy [38] and hip arthroplasty [46]. These procedures are performed by surgeons who specialize in orthopedics. In medical schools, for every general practice physician, standard tendon repair is of prime importance in fundamental surgical skills. Thus, it is necessary to develop tendon repair training [47].

22-ii) Highlight unanswered new questions, suggest future research

Highlight unanswered new questions, suggest future research.

	1	2	3	4	5	
subitem not at all important	○	○	○	○	◉	essential

清除選取

Does your paper address subitem 22-ii?

Copy and paste relevant sections from the manuscript (include quotes in quotation marks "like this" to indicate direct quotes from your manuscript), or elaborate on this item by providing additional information not in the ms, or briefly explain why the item is not applicable/relevant for your study

To our knowledge, this is the first study to adopt VR simulation for tendon repair training. It has been reported that the challenge of adopting VR simulation for regular curricula was due to the limited efficacy of VR as a learning tool [48]. To clarify the effectiveness, we demonstrated that the VR simulator was an effective tool in the acquisition of tendon repair in our blinded randomized trial. Modern VR simulations have a common disadvantage, which is the high cost. Clarke et al.[48] reported that individual simulators cost up to 6-figure sums. Our platform removed the cost for the students and was open to the public to maximize cost-effectiveness. To consider whether the VR simulator is an educational tool, the results have to be significantly differenced with positive feedback. If the VR group participants did not improve any surgical performance, the VR simulator was not considered as part of the regular training.

20) Trial limitations, addressing sources of potential bias, imprecision, and, if relevant, multiplicity of analyses

20-i) Typical limitations in ehealth trials

Typical limitations in ehealth trials: Participants in ehealth trials are rarely blinded. Ehealth trials often look at a multiplicity of outcomes, increasing risk for a Type I error. Discuss biases due to non-use of the intervention/usability issues, biases through informed consent procedures, unexpected events.

	1	2	3	4	5	
subitem not at all important	○	○	○	○	●	essential

清除選取

Does your paper address subitem 20-i? *

Copy and paste relevant sections from the manuscript (include quotes in quotation marks "like this" to indicate direct quotes from your manuscript), or elaborate on this item by providing additional information not in the ms, or briefly explain why the item is not applicable/relevant for your study

The follow-up period can only reflect the short-term effect of the VR simulator, which was a limitation of this study. The long-term effect on orthopedic specialists who practice on VR simulators could take years to evaluate.

21) Generalisability (external validity, applicability) of the trial findings

NPT: External validity of the trial findings according to the intervention, comparators, patients, and care providers or centers involved in the trial

21-i) Generalizability to other populations

Generalizability to other populations: In particular, discuss generalizability to a general Internet population, outside of a RCT setting, and general patient population, including applicability of the study results for other organizations

	1	2	3	4	5	
subitem not at all important	○	○	○	○	◉	essential

清除選取

Does your paper address subitem 21-i?

Copy and paste relevant sections from the manuscript (include quotes in quotation marks "like this" to indicate direct quotes from your manuscript), or elaborate on this item by providing additional information not in the ms, or briefly explain why the item is not applicable/relevant for your study

The VR simulator can provide a realistic surgical scenario, thus allowing students to train for a particular skill continuously or to master any unfamiliar procedures. According to the results, students learning from the VR simulator had significantly better scores than those learning from the traditional method with respect to the tendon repair technique. This finding may indicate that students using a VR simulator will be able to follow the whole operation more carefully and master the knowledge of the procedure in the future.

21-ii) Discuss if there were elements in the RCT that would be different in a routine application setting

Discuss if there were elements in the RCT that would be different in a routine application setting (e.g., prompts/reminders, more human involvement, training sessions or other co-interventions) and what impact the omission of these elements could have on use, adoption, or outcomes if the intervention is applied outside of a RCT setting.

	1	2	3	4	5	
subitem not at all important	○	○	○	○	◉	essential

清除選取

Does your paper address subitem 21-ii?

Copy and paste relevant sections from the manuscript (include quotes in quotation marks "like this" to indicate direct quotes from your manuscript), or elaborate on this item by providing additional information not in the ms, or briefly explain why the item is not applicable/relevant for your study

During the Cornonavirus-19 (COVID-19) pandemic, the use of technology in the medical education field has become a popular topic. Tendon repair is a procedure that requires senior professional surgeons; therefore, medical students and junior doctors may not practice enough to be able to perform suturing independently. The possible solution is for junior doctors to practice through the VR simulator, hence becoming more familiar with the procedure and more confident when performing it. The VR simulator can maximize a medical student's efficiency with respect to mastering this technique. It has been proven that the VR simulator in a simulation lab rather than in the operating room is a better practice method than the traditional classroom study in terms of the high flexibility of location [20]. Medical students or residents can perform tendon repair via the VR simulator before performing a formal operation. Using the VR simulator serves the purpose of shortening the operating time, reducing operation errors, and alleviating patients' postprocedure pain [49]. However, the expense of textbooks or teaching assistance when using the traditional method has no significant comparison with the investment in equipment for VR simulators.

OTHER INFORMATION

23) Registration number and name of trial registry

Does your paper address CONSORT subitem 23? *

Copy and paste relevant sections from the manuscript (include quotes in quotation marks "like this" to indicate direct quotes from your manuscript), or elaborate on this item by providing additional information not in the ms, or briefly explain why the item is not applicable/relevant for your study

Registration number and name of trial registry is ChiCTR2100046648; Chinese Clinical Trial Registry; A Primary Registry of International Clinical Trial Registry Platform, World Health Organization

24) Where the full trial protocol can be accessed, if available

Does your paper address CONSORT subitem 24? *

Cite a Multimedia Appendix, other reference, or copy and paste relevant sections from the manuscript (include quotes in quotation marks "like this" to indicate direct quotes from your manuscript), or elaborate on this item by providing additional information not in the ms, or briefly explain why the item is not applicable/relevant for your study

Full trial protocol can be accessed is on http://www.chictr.org.cn/index.aspx

25) Sources of funding and other support (such as supply of drugs), role of funders

Does your paper address CONSORT subitem 25? *

Copy and paste relevant sections from the manuscript (include quotes in quotation marks "like this" to indicate direct quotes from your manuscript), or elaborate on this item by providing additional information not in the ms, or briefly explain why the item is not applicable/relevant for your study

None sources of funding and other support.

X27) Conflicts of Interest (not a CONSORT item)

X27-i) State the relation of the study team towards the system being evaluated

In addition to the usual declaration of interests (financial or otherwise), also state the relation of the study team towards the system being evaluated, i.e., state if the authors/evaluators are distinct from or identical with the developers/sponsors of the intervention.

	1	2	3	4	5	
subitem not at all important	○	○	○	○	◉	essential

清除選取

Does your paper address subitem X27-i?

Copy and paste relevant sections from the manuscript (include quotes in quotation marks "like this" to indicate direct quotes from your manuscript), or elaborate on this item by providing additional information not in the ms, or briefly explain why the item is not applicable/relevant for your study

None conflicts of interest was presented.

About the CONSORT EHEALTH checklist

As a result of using this checklist, did you make changes in your manuscript? *

- ○ yes, major changes
- ○ yes, minor changes
- ◉ no

What were the most important changes you made as a result of using this checklist?

Title is the most important changes I made.

How much time did you spend on going through the checklist INCLUDING making changes in your manuscript *

I have spent 3 hours to finished.

As a result of using this checklist, do you think your manuscript has improved? *

- (●) yes
- ( ) no
- ( ) 其他：

Would you like to become involved in the CONSORT EHEALTH group?

This would involve for example becoming involved in participating in a workshop and writing an "Explanation and Elaboration" document

- (●) yes
- ( ) no
- ( ) 其他：

清除選取

Any other comments or questions on CONSORT EHEALTH

您的答案

STOP - Save this form as PDF before you click submit

To generate a record that you filled in this form, we recommend to generate a PDF of this page (on a Mac, simply select "print" and then select "print as PDF") before you submit it.

When you submit your (revised) paper to JMIR, please upload the PDF as supplementary file.

Don't worry if some text in the textboxes is cut off, as we still have the complete information in our database. Thank you!

Final step: Click submit !

Click submit so we have your answers in our database!

提交

請勿透過 Google 表格提交密碼。

Google 並未認可或建立這項內容。 舉報濫用情況 - 服務條款 - 私隱權政策

Google 表格
